# Inhibition of Iron Death by *Lycium barbarum* Polysaccharides Ameliorates Myocardial Injury in Sepsis: A Pharmacological Mechanism Study Based on the NRF2/HO‐1 Pathway

**DOI:** 10.1002/fsn3.70835

**Published:** 2025-09-17

**Authors:** Zesu Niu, Yi Hu, Long Yang, Xiaorui Meng, Mengfei Chen, Xue Bai, Ruxin Liu, Yujiao Tang, Liting Yang, Weiliang Xue

**Affiliations:** ^1^ The Third Clinical Medical School Ningxia Medical University Yinchuan China; ^2^ Department of Emergency Medicine, People's Hospital of Ningxia Hui Autonomous Region Ningxia Medical University Yinchuan China

**Keywords:** inflammation, *Lycium barbarum*, network pharmacology, sepsis‐induced myocardial injury

## Abstract

Sepsis remains a leading global cause of mortality, with sepsis‐induced myocardial injury (SIMI) being a critical determinant of clinical outcomes. *Lycium barbarum* L. (goji berry), a traditional Chinese herb, has demonstrated therapeutic potential in sepsis‐related organ injuries through multiple pathways. However, its role and mechanisms in SIMI remain unexplored. A network pharmacology approach identified candidate targets of *Lycium barbarum* via TCMSP and SwissTargetPrediction, intersected with sepsis/myocardial injury‐related targets from GeneCards. Protein–protein interaction (PPI) networks were constructed using STRING and analyzed via Cytoscape's cytoHubba plugin. Enriched pathways were explored through KEGG/GO analyses. Transcription factor prediction, molecular docking (AutoDock Vina), and molecular dynamics (MD) simulations (GROMACS) were performed for key targets. Experimental validation included in vivo LPS‐induced sepsis models and in vitro H9C2 cardiomyocyte assays. This study reveals that *Lycium barbarum* alleviates SIMI by modulating the NRF2/HO‐1 signaling pathway, positioning it as a novel therapeutic candidate for sepsis‐associated cardiac complications.

## Graphical abstract

This study elucidates that lipopolysaccharide‐binding protein (LBP) ameliorates sepsis‐induced myocardial injury by activating the nuclear factor erythroid 2‐related factor 2/heme oxygenase‐1 (NRF2/HO‐1) signaling pathway, thereby suppressing lipid peroxidation and ferroptosis.

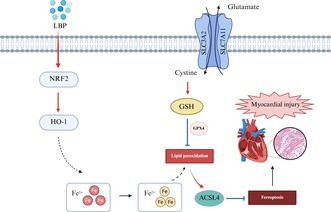

## 1 Introduction

Sepsis remains one of the leading causes of death worldwide and one of the most challenging global public health issues (Macdonald et al. 2017). The mortality rate of organ dysfunction caused by sepsis is approximately 40%, and cardiac dysfunction can further increase the mortality rate to around 80% (Bateman et al. 2016; Huang et al. 2021; Flierl et al. 2008). Sepsis‐Induced Cardiomyopathy (SIMI) is one of the most common and highest‐risk complications in septic patients, with an incidence rate of about 14% to 28.2% and a mortality rate ranging from 21.43% to 36.8% (Li et al. 2019; Bansal et al. 2022; Liang et al. 2021). Studies have shown that the mortality rate of SIMI patients with hospital stays longer than 10 days is significantly higher than that of patients without cardiac dysfunction (Lin et al. 2022). Currently, various anti‐inflammatory targeted therapies (such as TNF‐α antibodies and IL‐1 receptor antagonists) have not shown outstanding therapeutic effects for SIMI, making the development of effective treatments urgent (Sheng et al. 2023; Celes et al. 2013).

Goji berries ( *Lycium chinense* Miller) are also known as wolfberries. In traditional Chinese medicine, they are often used to boost the immune system and enhance vision (Wang et al. 2018; Tang et al. 2012). *Lycium barbarum* polysaccharide (LBP) is the most representative active component of goji berries and is believed to have numerous biological effects (Potterat 2010). Studies have indicated that LBP effectively controls the levels of inflammatory cytokines (TNF‐α and IL‐6) in RAW264.7 cell inflammation models, peritonitis mouse models, and heart failure rat models (Liu et al. 2021; Pop et al. 2020). These findings suggest that goji berries may play a role in mitigating sepsis‐related myocardial injury. However, the specific molecular mechanisms underlying these effects remain inadequately elucidated, indicating a significant gap in the current literature that warrants further investigation.

This study employs multi‐omics research methods to identify key active components and drug targets of goji berries through network pharmacology. We also validate the druggability of goji berries using molecular docking, molecular dynamics, and in vivo and in vitro experimental models. Additionally, we explore the potential therapeutic targets and related pathways involved in the treatment of sepsis‐induced myocardial injury by goji berries, and investigate the modes of cell death involved in the therapeutic process, providing insights into its potential therapeutic mechanisms.

In summary, this study aims to fill the knowledge gap regarding the therapeutic potential of goji berries in sepsis‐related myocardial injury. By elucidating the underlying mechanisms and identifying key molecular targets, we hope to develop more effective treatment strategies for this critical condition, ultimately improving patient outcomes and reducing the burden on healthcare systems.

## 2 Method

### 2.1 Main Reagents and Materials Used

LPS (lipopolysaccharide) used in this study was purchased from Sigma, USA, with the catalog number Cat No. 297‐473‐0. LBP ( *Lycium barbarum* polysaccharide) was purchased from Wolfberry Company, China. RIPA lysis buffer was purchased from Beyotime Biotechnology, China, with the catalog number P0013B. Trizol reagent, skim milk powder, and HE staining materials were also purchased from Beyotime Biotechnology, with the respective catalog numbers R0016, P0216‐1500 g, and C0105M. Antibodies used in Western Blot included GPX4, HO‐1, NRF2, ACSL4, and the internal reference GPPDH. Except for the SLC7A11 antibody, which was purchased from the USA (catalog number PA1‐16893), all other antibodies were purchased from Proteintech Group, China, with the respective catalog numbers 30388‐1‐AP, 10701‐1‐AP, 80593‐1‐RR, 22401‐1‐AP, and 10494‐1‐AP. The levels of IL‐1β in the Elisa experiment were detected using the Rat IL‐1β (Interleukin 1 Beta) ELISA Kit from Elabscience (catalog number E‐EL‐R0012c), and IL‐6 and TNF‐α were detected using the IL‐6 ELISA Kit (catalog number LM8K2868M) and TNF‐α ELISA Kit (catalog number LM8K1686M) from Lianshuo Biotech, respectively.

### 2.2 Experimental Animals and Cells

The mice selected for this study were C57BL/6N mice, all male, aged 10–12 weeks, with a weight of 18–22 g at the time of cage entry. The animal experiments were approved by the Ethics Committee of Ningxia Hui Autonomous Region People's Hospital, with the ethics number (2021)‐NZR‐098. The experimental cells were H9C2 cardiomyocytes obtained from the Cell Bank of the Typical Cultures Preservation Committee of the Chinese Academy of Sciences.

### 2.3 Identification of Goji Berry Targets and Sepsis‐Induced Myocardial Injury Targets

First, we searched for the active components of goji berries in the Traditional Chinese Medicine Systems Pharmacology Database and Analysis Platform (TCMSP) (https://www.tcmsp‐e.com/), using OB (oral bioavailability) > 30% and DL (drug‐likeness) > 0.18 as screening criteria to preliminarily identify active components with potential druggability, and obtained related drug targets of goji berries in TCMSP (Ru et al. 2014). Then, we searched for the SMILES IDs of these active components and further screened them on SwissADME (http://www.swissadme.ch/) to ensure that they can be absorbed by the gastrointestinal tract and have at least two “yes” in Druglikeness (Daina et al. 2017). Subsequently, we predicted the targets of these active components on SwissTargetPrediction, obtaining relevant target genes of goji berries (Gfeller et al. 2014). We took the union of these target genes and the druggable targets of goji berries in TCMSP to identify important targets related to goji berries. Then, we searched for targets related to sepsis and myocardial injury on the GeneCards website, taking the intersection of goji berry druggable targets, sepsis‐related targets, and myocardial injury‐related targets to obtain candidate targets (Stelzer et al. 2016).

### 2.4 Protein–Protein Interaction (PPI) Network Analysis and Cytoscape Screening of Hub Genes

We input the candidate targets into STRING. Click this URL for PPI analysis, selecting *Homo sapiens* as the species and setting the minimum required interaction score to 0.400 (Szklarczyk et al. 2015). Then, we input the results into Cytoscape software and used the CytoHubba plugin to screen the top 30 genes by Degree method (Shannon et al. 2003; Chin et al. 2014).

### 2.5 Gene Ontology (GO) Term Enrichment and Kyoto Encyclopedia of Genes and Genomes (KEGG) Pathway Analyses

We used the clusterProfiler package in R software to perform GO enrichment analysis and KEGG enrichment analysis, displaying the top 10 biological processes by Enrichment Score among the top 30 screened genes (Yu et al. 2012).

### 2.6 Screening of Upstream Transcription Factors of Target Proteins and Drug Molecular Docking and Pharmacokinetic Analysis

We used the TFTF package in R software to screen the upstream transcription factors of target proteins. We used CHEA3, ChIP‐Atlas, ENCODE, JASPAR Core database, GTRD, hTFtarget, FIMO, and the PWMEnrich package for prediction. The results were intersected, and an upset plot was drawn using the ggplot2 package (Wang 2024).

We obtained the mol numbers of active targets related to target genes from the TCMSP database, downloaded the sdf files of small molecules from the PubChem database, and searched for the protein structure pdb files of target genes in the PDB database. Then, we processed the protein files in Pymol software, performed molecular docking using Autodock software, and finally visualized the results using Discovery Studio software (Kim et al. 2019; Berman et al. 2003; Trott and Olson 2010). Molecular dynamics (MD) simulations were performed using the Gromacs2022 program, with small molecules using the GAFF force field, proteins using the AMBER14SB force field, and the TIP3P water model. The protein and small molecule ligand files were merged to construct the simulation system of the complex (Abraham et al. 2015). The simulation was conducted under constant temperature and pressure and periodic boundary conditions. During the MD simulation, all hydrogen bonds were constrained using the LINCS algorithm, with an integration step of 2 fs. Electrostatic interactions were calculated using the Particle‐Mesh Ewald (PME) method, with a cutoff value of 1.2 nm. The cutoff value for non‐bonded interactions was set to 10 Å, updated every 10 steps. The V‐rescale temperature coupling method was used to control the simulation temperature at 298 K, and the Berendsen method was used to control the pressure at 1 bar. At 298 K, 100 ps of NVT and NPT equilibrium simulations were performed, followed by 100 ns of MD simulation of the complex system, saving the conformation every 10 ps. After the simulation, the simulation trajectory was analyzed using VMD and Pymol, and the binding free energy between the protein and small molecule ligand was analyzed using the gmmpbsa program (Humphrey et al. 1996; Miller 3rd et al. 2012).

### 2.7 Establishment of Animal Model and Cell Model

Eighteen mice were randomly divided into three groups: control group, sepsis group, and treatment group, with six mice in each group. The sepsis mouse model was established by intraperitoneal injection of LPS at a concentration of 5 mg/kg (Shvilkina and Shapiro 2023; Gong et al. 2025). After 24 h, the activity and mental state of the mice were observed, and after another 24 h, the mice were anesthetized and euthanized. The apex blood of the mice was collected, and the heart specimens were fixed in 4% paraformaldehyde and then stored in a −80°C refrigerator. In the treatment group, after the first 24 h, LBP solution was injected intraperitoneally, and the same subsequent procedures as mentioned above were followed (Li et al. 2022). The control group was given the same amount of normal saline at both 24‐h intervals, and other steps were the same as above.

H9C2 cardiomyocytes were cultured in DMEM solution containing 10% fetal bovine serum and 1% penicillin solution, in an incubator at 37°C and 5% CO_2_. The cells were cultured, passaged, and collected using trypsin. The passaged H9C2 cells were divided into three groups: control group, sepsis group, and treatment group. When the cells reached 70% confluence in the culture dishes, 10 mg/mL of LPS was added to the sepsis and treatment groups. After 2 h of incubation, the cells of each group were centrifuged and collected for the next experiments.

### 2.8 Western Blot Analysis and Quantitative Polymerase Chain Reaction (qPCR) Analysis

First, H9C2 cells and mouse myocardial tissue samples were lysed, and proteins were extracted. The protein samples were quantified using the BCA method. The proteins were separated by SDS‐PAGE and transferred to PVDF membranes. The membranes were blocked with milk to reduce nonspecific binding and then incubated overnight at 4°C with specific primary antibodies. After washing, the membranes were incubated with labeled secondary antibodies to recognize the primary antibodies. After another round of washing to remove unbound secondary antibodies, the protein bands were visualized and recorded using chemiluminescence. ImageJ software was used to quantify the bands to assess the relative expression levels of target proteins. Statistical analysis of the data was performed using GraphPad Prism software, with one‐way ANOVA used to evaluate the differences in gene expression between different groups.

Total RNA was extracted from myocardial tissue and H9C2 cells using Trizol reagent, and its concentration and purity were measured using a nanophotometer. Using 1 μg of RNA as a template, cDNA was synthesized using M‐MLV reverse transcriptase. Each qPCR reaction system included the cDNA template, SYBR Green Master Mix, and specific primers (sequences are listed in Table 1). The reaction conditions were set to 95°C for 5 min of pre‐denaturation, followed by 40 cycles of 95°C for 10 s of denaturation and 60°C for 30 s of annealing and extension. The qPCR reactions were performed on a Roche LightCycler 480 instrument, and melting curve analysis was conducted to verify the specificity of the PCR products. The relative expression levels of target genes were calculated using the 2^−ΔΔCt^ method, with GAPDH as the reference gene for normalization. The statistical analysis methods used were the same as in the WB experiment.

**TABLE 1:** Sequences of qPCR primers for all target genes investigated in this study.

Name	Forward primer	Reverse primer
GPX4	AAGATCCAACCCAAGGGCAA	TCTTGTCGATGAGGAACTGTGG
HO‐1	CACGCATATACCCGCTACCT	CCAGAGTGTTCATTCGAGCA
SLC7A11	ATGGTCAGAAAGCCTGTTGTGT	TCAGCTGCACTTTCTCCTGC
NRF 2	GAGACGGCCATGACTGAT	TGTGGAACATCTGGTAGACGGC
ACSL4	CCTGAGGGGCTTGAAATTCAC	GTTGGTCTACTTGGAGGAACG
GAPDH	GACAACTTTGGCATCGTGGA	ATGCAGGGATGATGTTCTGG

### 2.9 Enzyme‐Linked Immunosorbent Assay (ELISA) for Inflammation Level Detection

Standard samples and test samples (mouse serum) were added to the ELISA plate and incubated at room temperature for 2 h. After washing, the appropriate enzyme‐labeled antibodies were added and incubated at room temperature for 1 h. After another round of washing, the substrate solution was added and incubated in the dark at room temperature for 15 min. The reaction was terminated by adding the stop solution, and the absorbance was measured at 450 nm using a microplate reader. The concentrations of TNF‐α, IL‐6, and IL‐1β in the samples were calculated based on the standard curve, and one‐way ANOVA was used to evaluate the differences in gene expression between different groups.

### 2.10 Histopathological Comparison of Myocardial Tissue Sections

Heart tissue frozen blocks were sectioned using a microtome, and the myocardial tissue sections were stained with hematoxylin and eosin. The stained sections were observed under a microscope at 20× magnification, and further image processing was performed using NDP.

## 3 Result

### 3.1 Systematic Identification of Goji Berry Bioactive Components and Their Therapeutic Targets in Sepsis‐Associated Myocardial Injury

**FIGURE 1:**
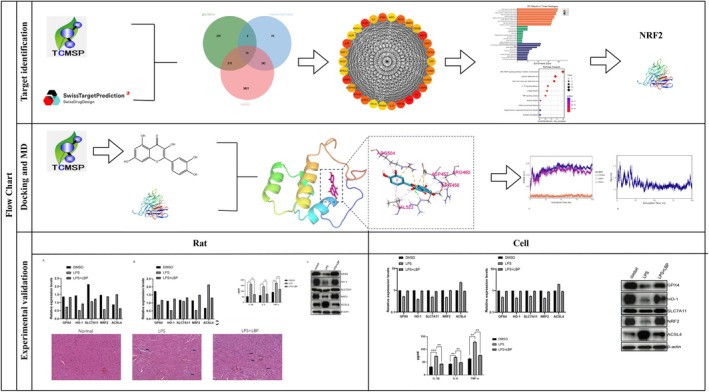
Network Pharmacology and Experimental Validation Flowchart.

To investigate the pharmacological basis of *Lycium barbarum* L. (goji berry) in sepsis‐related cardiac complications, we implemented a multi‐stage screening strategy for bioactive components. Initial screening of the TCMSP database employing stringent pharmacokinetic criteria (oral bioavailability ≥ 30% and drug‐likeness ≥ 0.18) identified 45 candidate compounds. Subsequent refinement using SwissADME's comprehensive druggability parameters (considering absorption and metabolic stability) yielded five optimal bioactive constituents: quercetin, mandenol, glycitein, atropine, and 7‐O‐methylluteolin‐6‐C‐beta‐glucoside_qt (Table S1 and Figure 1).

**FIGURE 2:**
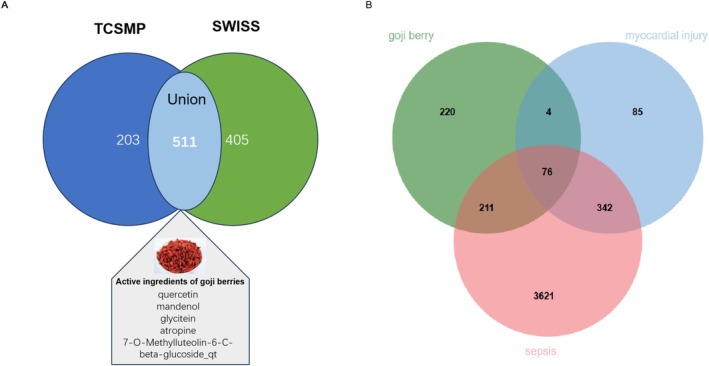
Venn Diagram of Goji Berry Targets and Sepsis‐Induced Myocardial Injury Drug Targets. Goji Berry Targets Venn Diagram. (A) The figure shows all the targets related to goji berries obtained by taking the union of predictions from the two tools, SwissTargetPrediction and TCSMP. (B) It demonstrates the targets through which goji berries may have potential therapeutic value for sepsis‐related myocardial injury.

Through target prediction analysis (TCMSP and SwissTargetPrediction platforms), we established 511 unique therapeutic targets for goji berry components after deduplication (Figure 2A). Parallel disease target mining from GeneCards revealed 4251 sepsis‐related targets and 508 myocardial injury‐associated genes. Intersectional analysis identified 76 critical hub genes at the nexus of goji berry pharmacology, sepsis pathogenesis, and myocardial injury mechanisms (Figure 2B), representing potential therapeutic targets for further investigation.

### 3.2 Construction of PPI Network and Screening of Hub Genes

**FIGURE 3:**
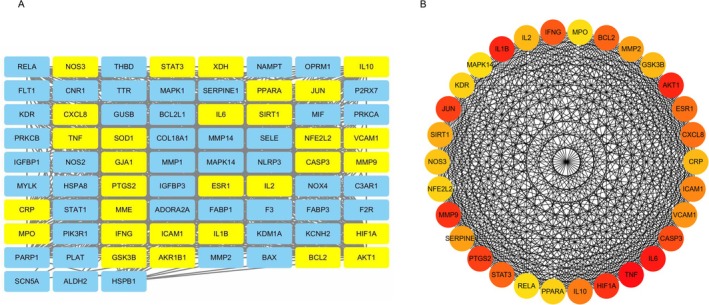
Use the Degree method to filter the top 30 genes by score. (A) A diagram showing the top 30 genes among the 76 genes, where yellow nodes represent hub genes and blue nodes represent less important genes. (B) An interaction diagram of the top 30 genes based on their scores, with darker colors indicating higher scores from the Degree method.

The 76 genes were subjected to PPI analysis using the STRING website. The results were then imported into Cytoscape software, where the CytoHubba plugin was used to score the included genes. The top 30 genes with the highest Degree scores were selected as hub genes for subsequent enrichment analysis (as shown in Figure 3).

### 3.3 Results of KEGG and GO Enrichment Analyses

**FIGURE 4:**
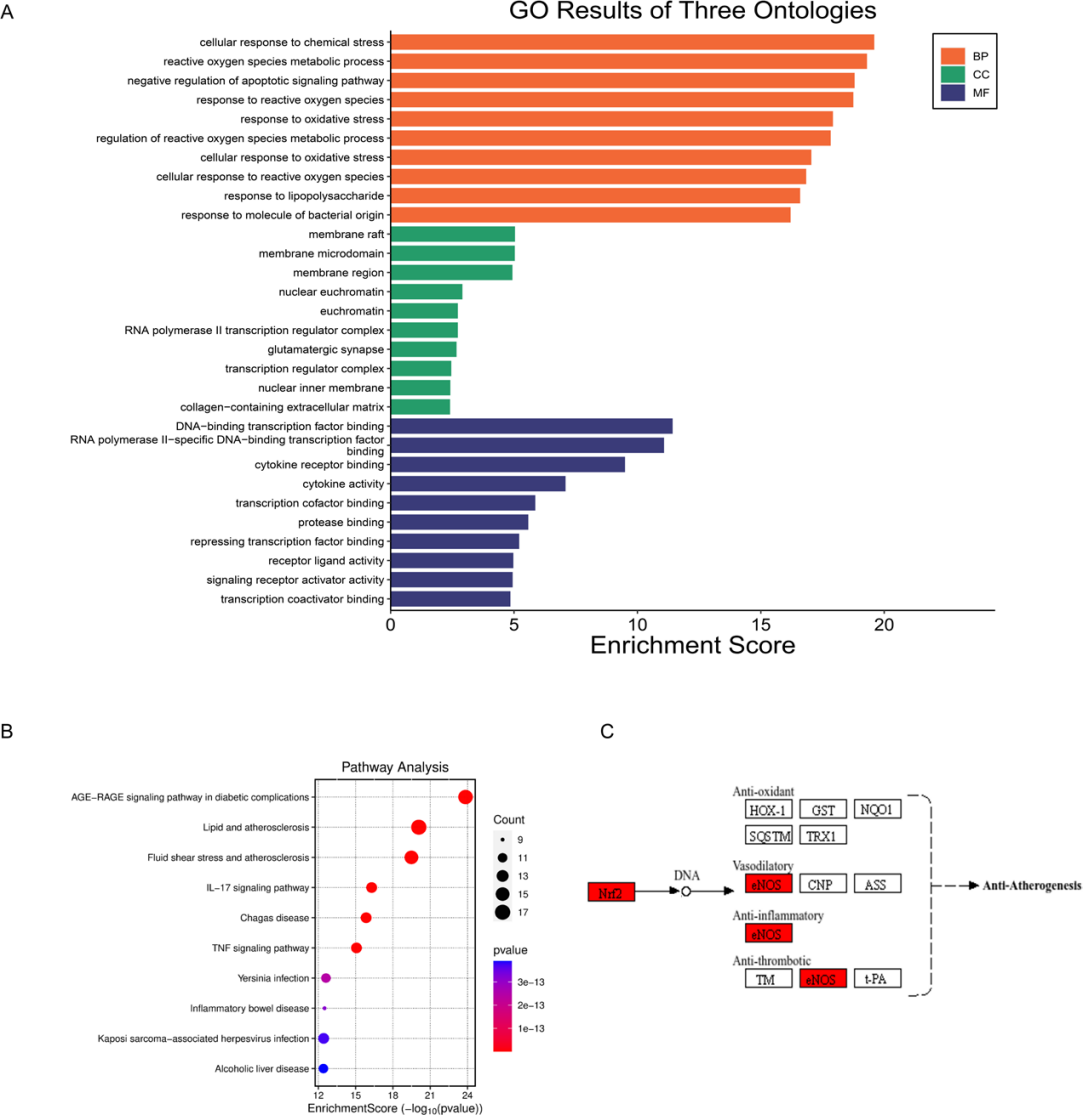
Bar chart of GO enrichment analysis, bubble chart of KEGG enrichment analysis, and mechanism diagram of NRF2 downstream pathways. (A) Enrichment analysis of hub genes conducted through the three layers of BP, CC, and MF, represented as a bar chart, where the length of the bars indicates the importance of the enriched biological processes, components, and pathways. (B) Bubble chart of KEGG enrichment analysis, where the size of the bubbles represents the number of enriched genes, and the color indicates the importance of the pathways. (C) Mechanism diagram of NRF2 downstream pathways, in which the antioxidant pathway shows significant importance in the GO enrichment analysis.

Subsequently, we performed KEGG and GO enrichment analyses on the 30 hub genes. The results showed significant enrichment of these genes in GO analysis, particularly in biological processes (BP), cellular components (CC), and molecular functions (MF) related to reactive oxygen species metabolism, oxidative stress response, and cell membranes closely associated with oxidative stress (see Figure 4A). In the KEGG enrichment analysis, these genes were notably associated with inflammation and infection‐related diseases and pathways (see Figure 4B). Upon reviewing relevant literature, we identified NRF2 as a crucial target among these 30 genes, due to its close association with reactive oxygen species and inflammatory processes. Furthermore, Figure 4C indicates that the NRF2 target may subsequently influence downstream HO‐1 protein, thereby affecting the antioxidant metabolism process.

### 3.4 Exploring Upstream Transcription Factors of NRF2

**FIGURE 5:**
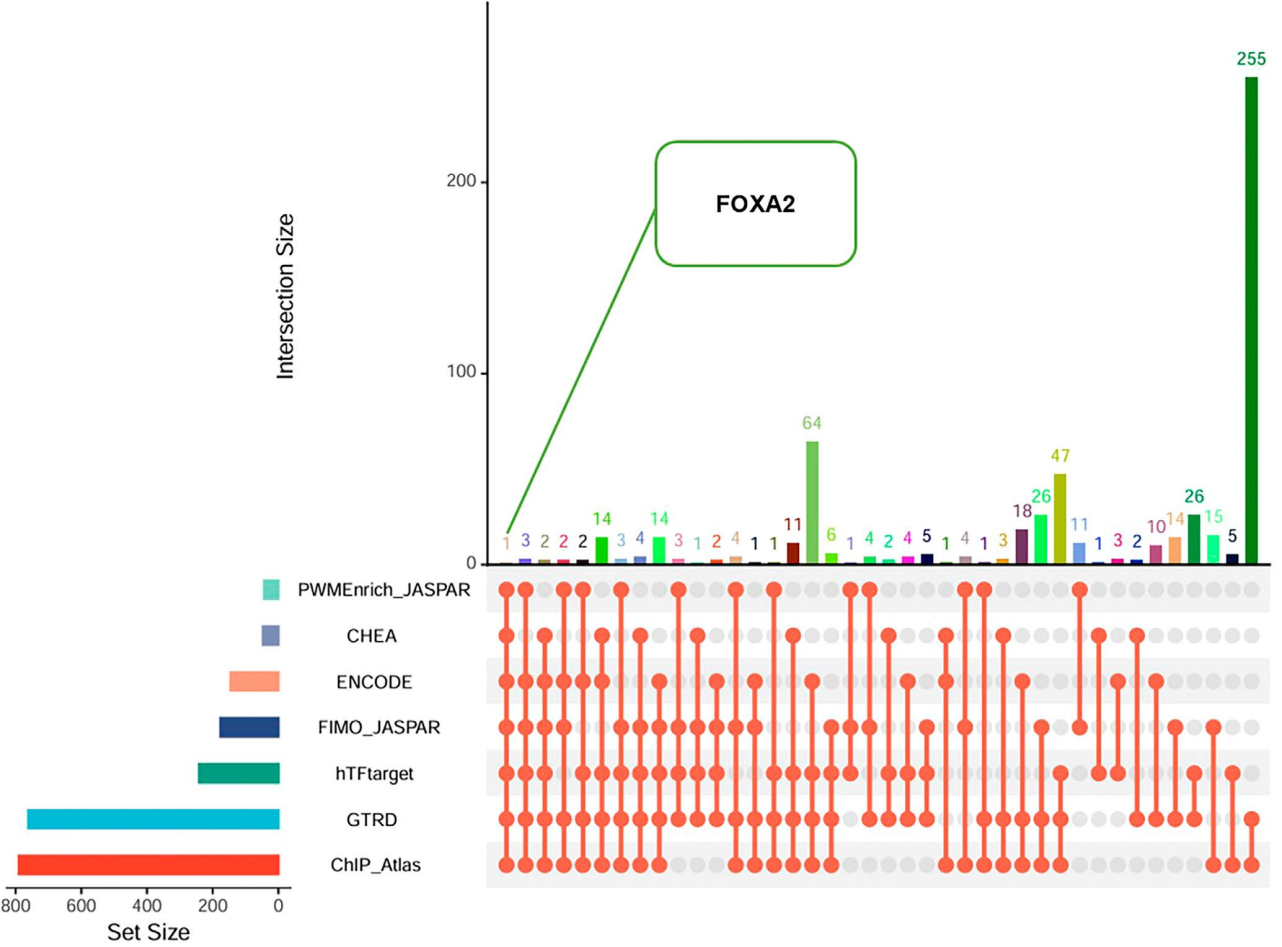
The figure shows the upset plot predicted by seven transcription factors, where the X‐axis represents the type of merging method, the Y‐axis represents the number of transcription factors, and the text on the left side of the axes represents the names of the methods.

We utilized seven transcription factor prediction methods to predict the transcription factors of NRF2 (see Table S2 for details). By intersecting the results, we found that FOXA2 appeared in all seven prediction methods, making it a noteworthy subject for further investigation (see Figure 5).

### 3.5 Molecular Docking and Molecular Dynamics Analysis of NRF2‐Related Active Components in Goji Berry

**FIGURE 6:**
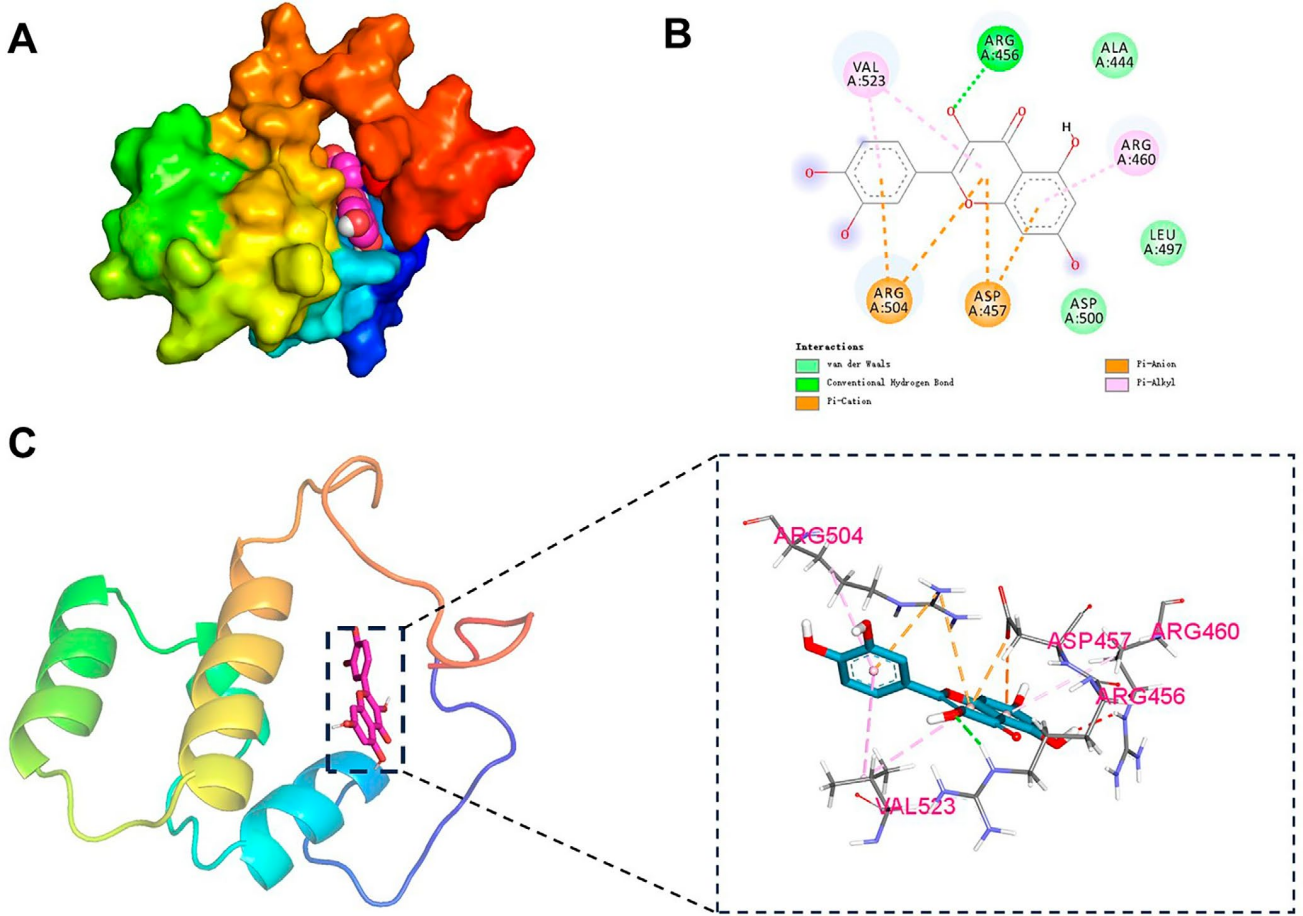
Molecular docking diagram of NRF2 and quercetin. (A) 3D structure diagram of the molecular docking. (B) 2D diagram of the small molecule docking, where the nodes surrounding the small molecule represent the connected proteins, and different colors indicate different types of interactions. (C) Overall diagram of the molecular docking and a local view.

**FIGURE 7:**
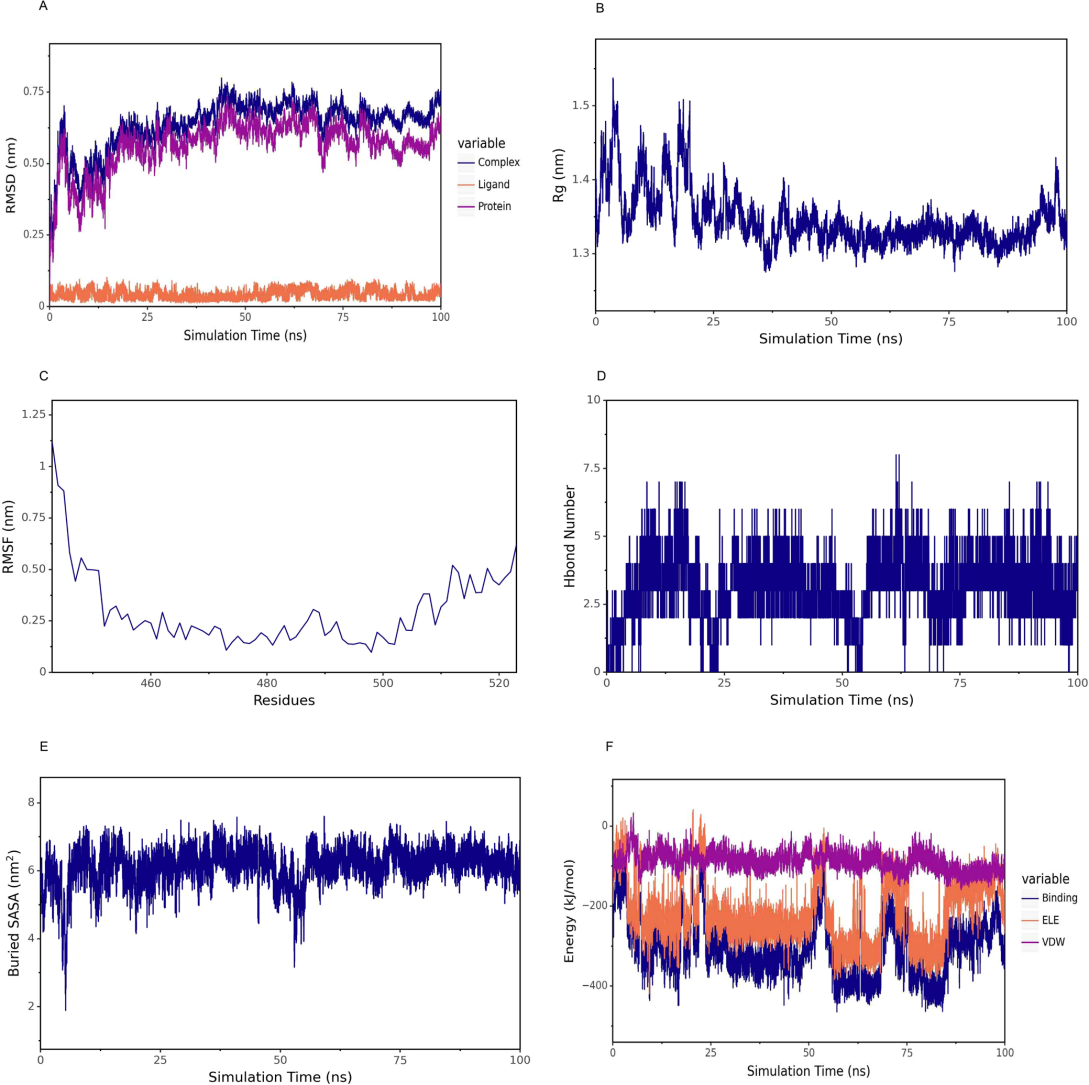
Molecular dynamics results figures. (A) RMSD of the complex, protein, and small molecule ligand. (B) Rg of the complex. (C) RMSF of the protein in the complex. (D) Hydrogen bond number. (E) Buried solvent accessible surface area (SASA) between the small molecule and the protein. (F) Binding energy (Van der Waals and Electrostatic) between the small molecule and the protein.

Through the TCMSP database, we found that among the five active components with drug‐like properties, only quercetin has a target relationship with NRF2. Therefore, we selected quercetin for molecular docking with NRF2 (see Table S1). The results showed that the affinity between quercetin and NRF2 was −6.7 kcal/mol, indicating a certain binding ability between them (as shown in Figure 6). In subsequent molecular dynamics analysis, RMSD analysis results showed that the RMSD of quercetin, an active component of goji berry, fluctuated synchronously with the RMSD of NRF2 protein, indicating that the fluctuation of the complex's RMSD was caused by the protein itself. The RMSD of quercetin remained stable during the simulation, indicating that the structure of the complex was generally stable (as shown in Figure 7A). The Radius of Gyration (Rg) analysis suggested that the Rg of the complex gradually decreased and stabilized during the simulation, indicating that the complex structure remained stable (as shown in Figure 7B). Root Mean Square Fluctuation (RMSF) displayed the fluctuations of each atom or residue to identify important dynamic regions (as shown in Figure 7C). Hydrogen bonds are an important interaction force in the binding of proteins and ligands. Hydrogen bonds are related to electrostatic interactions and can reflect the strength of electrostatic interactions. Figure 7D shows that the number of hydrogen bonds between the small molecule and the protein fluctuated, generally maintaining between 2 and 5. Buried Solvent Accessible Surface Area (Buried SASA) is used to evaluate which parts of a molecule or molecular complex are buried inside and not directly accessible to the solvent. The larger the Buried SASA value, the stronger the interaction between molecules and the larger the contact area. Figure 7E shows that Buried SASA gradually stabilized, indicating that the contact area between quercetin and NRF2 gradually stabilized, and their binding gradually stabilized. Simultaneously, without considering solvation, we calculated the van der Waals forces and electrostatic interactions between the small molecule and the protein in the complex to analyze the changes in binding force during the simulation. VDW represents van der Waals forces and hydrophobic interactions, ELE represents electrostatic interactions, and Binding is the sum of VDW and ELE, representing the binding energy between the small molecule and the protein without considering solvation effects. Figure 7F shows that the electrostatic interaction force ELE in the complex fluctuated during the simulation, due to the conformational adjustment of the small molecule binding to the protein. However, this did not affect the overall stability of the small molecule and protein binding, which remained stable. In summary, we found that the binding between quercetin and NRF2 is relatively stable.

### 3.6 HE Staining Results

**FIGURE 8:**
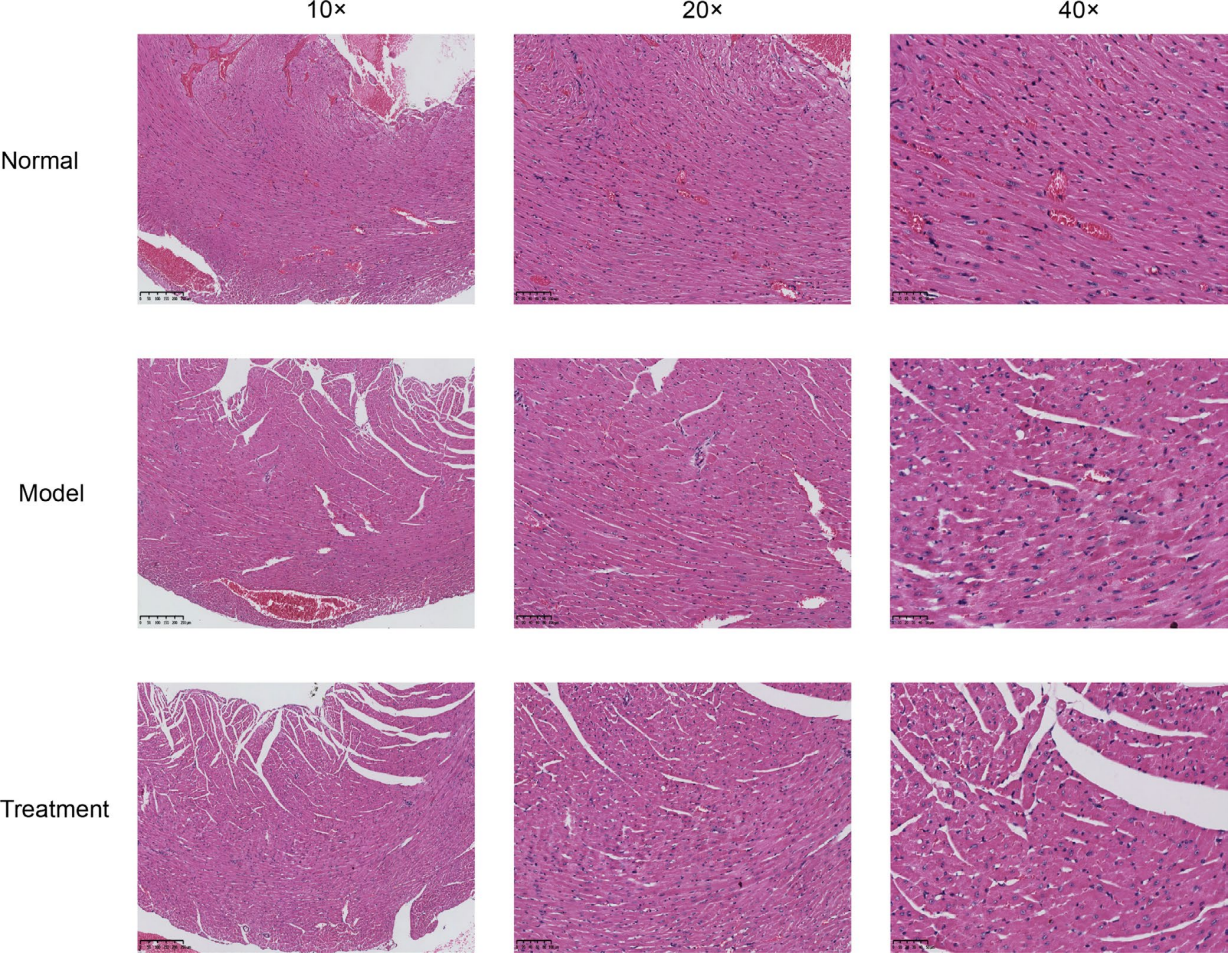
Histopathological sections of myocardial tissue from each group of mice. Representative H&E‐stained tissue sections from different groups at magnifications of 10×, 20×, and 40×. ‘Normal’ group displayed regular tissue architecture with well‐organized cellular alignment and no evident pathological features. In the ‘Model’ group, noticeable histopathological alterations were observed, including disordered cellular arrangement, increased fibrous tissue deposition, and inflammatory cell infiltration. The ‘Treatment’ group showed marked improvement in tissue morphology, with partial restoration of cellular organization and a significant reduction in fibrous tissue and inflammatory infiltration compared to the ‘Model’ group.

HE staining of pathological sections indicated the effect of LBP on myocardial tissue pathological changes in septic mice. In Figure 8A, the control group, representing healthy mice, showed normal tissue structure with no significant abnormalities. In contrast, after LPS induction, Figure 8B displayed significant inflammatory cell infiltration and tissue structure destruction (as indicated by arrows). Subsequently, Figure 8C, representing the treatment group, showed that compared to the model group, the occurrence of inflammation and the disruption of tissue integrity were significantly reduced. To further quantify the pathological section conditions of each group, two researchers independently evaluated the results of the sections according to relevant pathological scoring criteria, and the findings were consistent with the aforementioned assessment (see Table S3 for details).

### 3.7 qPCR and Western Blot Analysis Validate That Goji Berry Mitigates Septic Myocardial Injury by Activating the NRF2/HO1 Pathway

**FIGURE 9:**
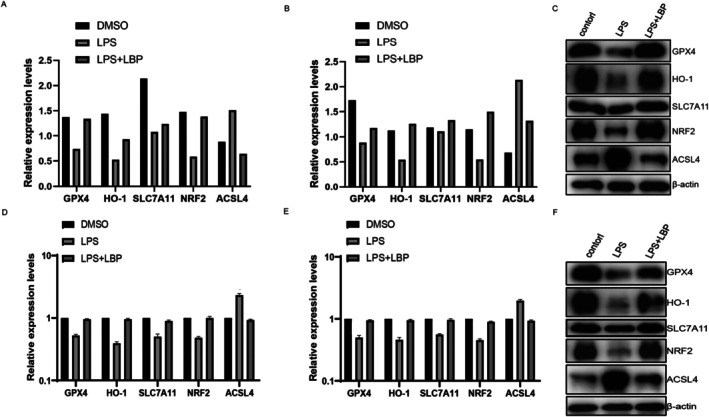
Histograms of WB and qPCR results for key proteins in the NRF2/HO‐1 pathway. (A, B) Comparison of protein‐level expressions of GPX, HO‐1, SLC7A11, NRF2, and ACSL4 among the control group, sepsis group, and treatment group in both cellular and animal models. (D, E) Comparison of mRNA‐level expressions of GPX, HO‐1, SLC7A11, NRF2, and ACSL4 among the control group, sepsis group, and treatment group in both cellular and animal models. (C) WB band images for the cellular model. (F) WB band images for the animal model.

**FIGURE 10:**
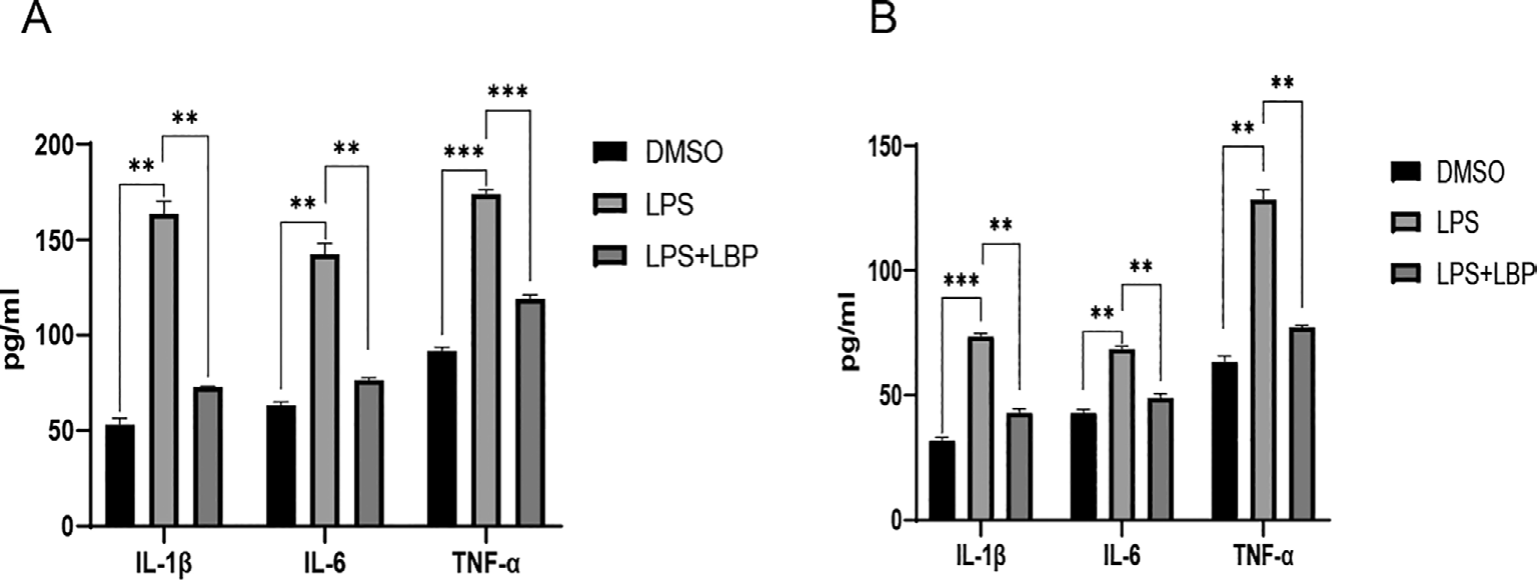
Histograms of inflammatory factors measured by ELISA in cellular and animal models. (A) Cellular model. (B) Animal model.*p < 0.05, **p < 0.01, and ***p < 0.005.

As a central regulator of antioxidation, the inactivation, inhibition, or knockdown of Nrf2 enhances ferroptosis in cells. Iron metabolism proteins and ferritin, regulated by Nrf2, are involved in ferroptosis. Figure 9A–C show that, compared to the control group, the model group exhibited lower mRNA and protein expression levels of Nrf2, HO‐1, GPX4, and SLC7A11, whereas ACSL4 mRNA and protein expression levels were higher. After treatment with LBP, the mRNA and protein expression levels of Nrf2, HO‐1, GPX4, and SLC7A11 were significantly increased, while the mRNA and protein expression levels of ACSL4 were reduced. This indicates that LBP can activate the Nrf2/HO‐1 signaling pathway and may also inhibit ferroptosis. Specific statistical data are provided in Table S4 (Figure 10).

### 3.8 ELISA Results Indicate the Anti‐Inflammatory Effects of Goji Berry in Septic Myocardial Injury

We found that, in both animal and cell models, the levels of IL‐1β, IL‐6, and TNF‐α were significantly higher in the model group compared to the control group. However, after LBP treatment, the levels of IL‐1β, IL‐6, and TNF‐α were significantly lower than those in the model group.

## 4 Discussion

Septic cardiomyopathy remains a lethal complication in critical care, with unmet therapeutic needs despite standard sepsis management (Merdji et al. 2021). Our study identifies *Lycium barbarum* polysaccharides (LBP) as a promising intervention, with quercetin emerging as its bioactive core component for mitigating myocardial injury (Ma et al. 2023; Sang et al. 2022; Ding et al. 2024).

Mechanistically, KEGG/GO enrichment and molecular docking revealed that LBP enhances antioxidative responses via NRF2 activation—a pathway critically implicated in sepsis‐induced myocardial dysfunction (Fang et al. 2024; Jin et al. 2020). The high‐affinity quercetin‐NRF2 binding, validated by molecular dynamics, underscores quercetin's potential as a lead compound for targeted therapy (Kumar et al. 2017). In vivo/in vitro data further confirmed NRF2/HO‐1 pathway upregulation as the primary protective mechanism, with concurrent downregulation of ferroptosis marker ACSL4 suggesting inhibition of this cell death modality (Lu et al. 2021; Liu et al. 2024).

Myocardial injury results from impaired microcirculation, direct myocardial suppression, and mitochondrial dysfunction, with oxidative stress playing a pivotal role (Lukić et al. 2024). In septic cardiomyopathy, the excessive inflammatory response triggered by infection induces lipid peroxidation in cardiomyocytes, leading to structural disruption and functional loss of critical organelles such as mitochondria and the endoplasmic reticulum, ultimately culminating in irreversible myocardial necrosis. Notably, Wu et al. demonstrated that USP13 overexpression mitigates apoptosis, oxidative stress, and pro‐inflammatory cytokine production by modulating the Nrf2 pathway, thereby conferring cardioprotection (Wu et al. 2023). This finding aligns closely with our current study, further solidifying NRF2 as a key therapeutic target in septic cardiomyopathy.

Recent evidence highlights a critical interplay between NRF2 and ferroptosis, suggesting that the “NRF2‐lipid peroxidation‐ferroptosis” axis may represent a novel therapeutic avenue for sepsis. For instance, Wang et al. showed that irisin protects against sepsis‐associated encephalopathy by activating the Nrf2/GPX4 signaling cascade to suppress ferroptosis (Wang et al. 2022). Similarly, Lai et al. demonstrated that uridine attenuates sepsis‐induced acute lung injury by inhibiting macrophage ferroptosis, with NRF2 identified as a pivotal regulatory node (Lai et al. 2023).

In this study, quercetin was identified as a bioactive component of *Lycium barbarum* polysaccharides, with its drug‐likeness further validated through molecular docking and dynamics simulations (Alizadeh and Ebrahimzadeh 2022). Clinically, quercetin has shown promise in multiple randomized controlled trials (RCTs). Shi et al. reported that quercetin significantly reduces plasma uric acid levels (−26.5 μmol/L) in hyperuricemia patients without affecting fasting glucose, urinary uric acid excretion, or blood pressure (Shi and Williamson 2016). More importantly, a Ukrainian RCT demonstrated that intravenous quercetin administration correlates with reduced incidence of reperfusion‐related myocardial hemorrhage in myocardial infarction patients (Kozhukhov et al. 2024). These findings underscore the favorable clinical prospects of quercetin.

There are some limitations to this study that should be acknowledged. First, the lack of clinical validation raises concerns about the applicability of the findings to human subjects. Although in vivo and in vitro models provided important insights, the relatively small sample size in animal experiments may limit the generalizability of the results. Additionally, potential batch effects and variability in the multiple datasets used during bioinformatics analysis could introduce biases, affecting the robustness of target identification. These limitations underscore the need for further research, particularly clinical trials, to confirm the therapeutic potential of Goji berry polysaccharides in sepsis‐associated myocardial injury.

In conclusion, this study elucidates the mechanisms by which Goji berry may exert protective effects against sepsis‐associated myocardial injury, emphasizing the activation of the NRF2/HO‐1 signaling pathway as a key intervention route. The protective effect of Goji berry may be attributed to its rich quercetin content, which, by activating the crucial target NRF2, provides a protective mechanism against sepsis‐induced myocardial injury through the inhibition of ferroptosis. Future research should focus on validating these findings in clinical settings to translate the observed benefits into effective treatments for patients with sepsis and its cardiovascular complications.

## 5 Conclusion

In conclusion, our study reveals that *Lycium barbarum* L. ameliorates sepsis‐induced myocardial injury by suppressing ferroptosis through NRF2/HO‐1 pathway activation. Notably, quercetin was identified as the predominant bioactive compound responsible for these cardioprotective effects, demonstrating strong binding affinity with NRF2 in both molecular docking and molecular dynamics simulations. Furthermore, we discovered FOXA2 as a potential upstream transcriptional regulator of NRF2, suggesting a novel regulatory axis (FOXA2/NRF2/HO‐1) for targeted intervention in septic cardiomyopathy.

## Author Contributions

**Mengfei Chen, Weiliang Xue:** conceived and designed. **Zesu Niu, Yi Hu**, and **Xue Bai:** conducted the network pharmacology and bioinformatics analyses. **Zesu Niu, Yi Hu, Liting Yang, Ruxin Liu, Long Yang**, and **Mengfei Chen:** participated in the animal experiments. **Zesu Niu, Yi Hu, Xue Bai, Xiaorui Meng**, and **Liting Yang:** cell experiments. **Zesu Niu, Yi Hu:** drafted the manuscript. **Mengfei Chen, Weiliang Xue:** contributed to the experimental design and revised the manuscript. **Yujiao Tang:** supervision. All authors reviewed and approved the final manuscript.

## Ethics Statement

This study was approved by the Medical Ethics Committee of Ningxia Hui Autonomous Region People's Hospital (approval number: (2021)‐NZR‐098).

## Conflicts of Interest

The authors declare no conflicts of interest.

## Supporting information


**Table S1:** fsn370835‐sup‐0001‐TableS1.docx.

**Table S2:** fsn370835‐sup‐0002‐TableS2.docx.

**Table S3:** fsn370835‐sup‐0003‐TableS3.docx.

**Table S4:** fsn370835‐sup‐0004‐TableS4.docx.

## Data Availability

The datasets generated and analyzed during the current study are available in the supporting information files or upon reasonable request from the corresponding author. For the network pharmacology analysis, the target prediction datasets were obtained from TCMSP (https://old.tcmsp‐e.com/) and SwissTargetPrediction (http://www.swisstargetprediction.ch/), while disease‐associated targets were sourced from GeneCards (https://www.genecards.org/, version 5.16). Protein–protein interaction (PPI) network data were retrieved from STRING (https://string‐db.org/), all of which are publicly accessible via their respective platforms. Processed data, including the intersection of targets and core gene lists, are provided in the supporting information of this article. For molecular docking and dynamics analyses, protein structures were obtained from the RCSB Protein Data Bank (PDB; https://www.rcsb.org/) using the specified PDB IDs. The ligand structure of quercetin was sourced from PubChem (https://pubchem.ncbi.nlm.nih.gov/). The raw molecular docking scores and molecular dynamics simulation trajectories (GROMACS output files) are available upon request. Experimental data, including raw Western blot (WB) images and optical density quantification results, as well as quantitative polymerase chain reaction (qPCR) data (Ct values and relative expression calculations), are provided in the supporting information files. Enzyme‐linked immunosorbent assay (ELISA) data (raw absorbance readings for IL‐1β, IL‐6, and TNF‐α, along with cytokine concentration calculations) and histopathological images are archived by the authors. For inquiries regarding data access, please contact the corresponding author, Professor Wei‐Liang Xue (19995350081@163.com). ORCID: https://orcid.org/0009‐0004‐7440‐3782.
