# Supplementary material for: Inhibition of Iron Death by Lycium barbarum Polysaccharides Ameliorates Myocardial Injury in Sepsis: A Pharmacological Mechanism Study Based on the NRF2/HO‐1 Pathway
Source: Food Sci Nutr. 2025 Sep 17;13(9):e70835. doi: 10.1002/fsn3.70835 (PMC12441308; doi:10.1002/fsn3.70835)
Supplement: Supplementary file 1 — Table S1: fsn370835‐sup‐0001‐TableS1.docx. [file FSN3-13-e70835-s003.docx]

| MOLID | moleculename | target_name | UniProtAC | GeneNames | target_ID | drugbank_ID |
| --- | --- | --- | --- | --- | --- | --- |
| MOL000098 | quercetin | Prostaglandin G/H synthase 1 | P23219 | PTGS1 | 6 | 20 |
| MOL000098 | quercetin | Androgen receptor | P10275 | AR | 48 | 146 |
| MOL000098 | quercetin | Peroxisome proliferator activated receptor gamma | P37231 | PPARG | 78 | 238 |
| MOL000098 | quercetin | Prostaglandin G/H synthase 2 | P35354 | PTGS2 | 94 | 290 |
| MOL000098 | quercetin | Heat shock protein HSP 90 | P07900 | HSP90AA1 | 444 | 1939 |
| MOL000098 | quercetin | Phosphatidylinositol-4,5-bisphosphate 3-kinase catalytic subunit, gamma isoform | P48736 | PIK3CG | 491 | 2404 |
| MOL000098 | quercetin | Nuclear receptor coactivator 2 | Q15596 | NCOA2 | 3276 | 6241 |
| MOL000098 | quercetin | Dipeptidyl peptidase IV | P27487 | DPP4 | 332 | 952 |
| MOL000098 | quercetin | Aldose reductase | P15121 | AKR1B1 | 288 | 822 |
| MOL000098 | quercetin | Trypsin-1 | P07477 | PRSS1 | 2928 | 3176 |
| MOL000098 | quercetin | DNA topoisomerase II | P11388 | TOP2A | 287 | 817 |
| MOL000098 | quercetin | Thrombin | P00734 | F2 | 17 | 54 |
| MOL000098 | quercetin | Potassium voltage-gated channel subfamily H member 2 | Q12809 | KCNH2 | 37 | 101 |
| MOL000098 | quercetin | Sodium channel protein type 5 subunit alpha | Q14524 | SCN5A | 70 | 220 |
| MOL000098 | quercetin | Coagulation factor Xa | P00742 | F10 | 79 | 239 |
| MOL000098 | quercetin | Beta-2 adrenergic receptor | P07550 | ADRB2 | 261 | 766 |
| MOL000098 | quercetin | Stromelysin-1 | P08254 | MMP3 | 441 | 1926 |
| MOL000098 | quercetin | mRNA of PKA Catalytic Subunit C-alpha | P17612 | PRKACA | 699 | 6263 |
| MOL000098 | quercetin | Coagulation factor VII | P08709 | F7 | 123 | 369 |
| MOL000098 | quercetin | Nitric-oxide synthase, endothelial | P29474 | NOS3 | 95 | 291 |
| MOL000098 | quercetin | Retinoic acid receptor RXR-alpha | P19793 | RXRA | 158 | 459 |
| MOL000098 | quercetin | Acetylcholinesterase | P22303 | ACHE | 165 | 474 |
| MOL000098 | quercetin | Gamma-aminobutyric acid receptor subunit alpha-1 | P14867 | GABRA1 | 309 | 872 |
| MOL000098 | quercetin | Amine oxidase [flavin-containing] B | P27338 | MAOB | 565 | 3939 |
| MOL000098 | quercetin | Epidermal growth factor receptor | P00533 | EGFR | 298 | 844 |
| MOL000098 | quercetin | Vascular endothelial growth factor A | P15692 | VEGFA | 740 | 183 |
| MOL000098 | quercetin | Apoptosis regulator Bcl-2 | P10415 | BCL2 | 86 | 273 |
| MOL000098 | quercetin | Urokinase-type plasminogen activator | P00749 | PLAU | 346 | 1074 |
| MOL000098 | quercetin | 72 kDa type IV collagenase | P08253 | MMP2 | 238 | 707 |
| MOL000098 | quercetin | Mitogen-activated protein kinase 1 | P28482 | MAPK1 | 354 | 1176 |
| MOL000098 | quercetin | Pro-epidermal growth factor | P01133 | EGF | 734 | 1278 |
| MOL000098 | quercetin | Retinoblastoma-associated protein | P06400 | RB1 | 2915 | 3846 |
| MOL000098 | quercetin | Tumor necrosis factor | P01375 | TNF | 265 | 777 |
| MOL000098 | quercetin | Transcription factor AP-1 | P05412 | JUN | 414 | 1629 |
| MOL000098 | quercetin | Interleukin-6 | P05231 | IL6 | 351 | 1159 |
| MOL000098 | quercetin | Cellular tumor antigen p53 | P04637 | TP53 | 646 | 5788 |
| MOL000098 | quercetin | NADPH--cytochrome P450 reductase | P16435 | POR | 744 | 4120 |
| MOL000098 | quercetin | Ornithine decarboxylase | P11926 | ODC1 | 153 | 449 |
| MOL000098 | quercetin | Xanthine dehydrogenase/oxidase | P47989 | XDH | 568 | 3947 |
| MOL000098 | quercetin | DNA topoisomerase 1 | P06612 | topA | 1293 | 3552 |
| MOL000098 | quercetin | Superoxide dismutase [Cu-Zn] | P00441 | SOD1 | 597 | 4152 |
| MOL000098 | quercetin | Interstitial collagenase | P03956 | MMP1 | 353 | 1167 |
| MOL000098 | quercetin | Cell division control protein 2 homolog | P06493 | CDK1 | 431 | 1771 |
| MOL000098 | quercetin | 78 kDa glucose-regulated protein | P11021 | HSPA5 | 2963 | 1847 |
| MOL000098 | quercetin | Acetyl-CoA carboxylase 1 | Q13085 | ACACA | 231 | 690 |
| MOL000098 | quercetin | Heme oxygenase 1 | P72849 | pbsA1 | 2132 | 3391 |
| MOL000098 | quercetin | Cytochrome P450 3A4 | P08684 | CYP3A4 | 621 | 4512 |
| MOL000098 | quercetin | Cytochrome P450 1A2 | P05177 | CYP1A2 | 724 | 4200 |
| MOL000098 | quercetin | Tissue factor | P13726 | F3 | 466 | 2139 |
| MOL000098 | quercetin | Gap junction alpha-1 protein | P17302 | GJA1 | 436 | 1827 |
| MOL000098 | quercetin | Interleukin-1 beta | P01584 | IL1B | 418 | 1654 |
| MOL000098 | quercetin | C-C motif chemokine 2 | P13500 | CCL2 | 417 | 1649 |
| MOL000098 | quercetin | E-selectin | P16581 | SELE | 427 | 1756 |
| MOL000098 | quercetin | Vascular cell adhesion protein 1 | P19320 | VCAM1 | 440 | 1908 |
| MOL000098 | quercetin | Prostaglandin E2 receptor EP3 subtype | P43115 | PTGER3 | 593 | 4131 |
| MOL000098 | quercetin | Estrogen sulfotransferase | P49888 | SULT1E1 | 781 | 1275 |
| MOL000098 | quercetin | Maltase-glucoamylase, intestinal | O43451 | MGAM | 318 | 929 |
| MOL000098 | quercetin | Interleukin-2 | P60568 | IL2 | 3978 | 1575 |
| MOL000098 | quercetin | Tissue-type plasminogen activator | P00750 | PLAT | 434 | 1792 |
| MOL000098 | quercetin | Thrombomodulin | P07204 | THBD | 457 | 2021 |
| MOL000098 | quercetin | Collagen alpha-1(I) chain | P02452 | COL1A1 | 731 | 28 |
| MOL000098 | quercetin | Interferon gamma | P01579 | IFNG | 365 | 1253 |
| MOL000098 | quercetin | Arachidonate 5-lipoxygenase | P09917 | ALOX5 | 88 | 275 |
| MOL000098 | quercetin | Myeloperoxidase | P05164 | MPO | 428 | 1757 |
| MOL000098 | quercetin | Glutathione S-transferase P | P09211 | GSTP1 | 733 | 904 |
| MOL000098 | quercetin | NAD(P)H dehydrogenase [quinone] 1 | P15559 | NQO1 | 470 | 2157 |
| MOL000098 | quercetin | Aryl hydrocarbon receptor | P35869 | AHR | 3204 | 6220 |
| MOL000098 | quercetin | Collagen alpha-1(III) chain | P02461 | COL3A1 | 2885 | 3853 |
| MOL000098 | quercetin | DNA gyrase subunit B | P0A0K8 | gyrB | 1366 | 4150 |
| MOL000098 | quercetin | Insulin receptor | P06213 | INSR | 11 | 36 |
| MOL000098 | quercetin | Prostatic acid phosphatase | P15309 | ACP3 | 2998 | 1859 |
| MOL000098 | quercetin | Cathepsin D | P07339 | CTSD | 363 | 1243 |
| MOL000098 | quercetin | Serum paraoxonase/arylesterase 1 | P27169 | PON1 | 357 | 1198 |
| MOL000098 | quercetin | Glutathione S-transferase Mu 1 | P09488 | GSTM1 | 2952 | 896 |
| MOL000098 | quercetin | Glutathione S-transferase Mu 2 | P28161 | GSTM2 | 3688 | 2165 |
| MOL000098 | quercetin | Transcription factor p65 | Q04206 | RELA | 4565 | 1 |
| MOL000098 | quercetin | RAC-alpha serine/threonine-protein kinase | P31749 | AKT1 | 4490 | 1 |
| MOL000098 | quercetin | G1/S-specific cyclin-D1 | P24385 | CCND1 | 4214 | 1 |
| MOL000098 | quercetin | Bcl-2-like protein 1 | O43521 | BCL2L11 | 4054 | 1 |
| MOL000098 | quercetin | Cyclin-dependent kinase inhibitor 1 | P38936 | CDKN1A | 4141 | 1 |
| MOL000098 | quercetin | Caspase-9 | P55211 | CASP9 | 4090 | 1 |
| MOL000098 | quercetin | Matrix metalloproteinase-9 | P14780 | MMP9 | 4334 | 1 |
| MOL000098 | quercetin | Interleukin-10 | P22301 | IL10 | 4292 | 1 |
| MOL000098 | quercetin | Caspase-3 | P42574 | CASP3 | 4087 | 1 |
| MOL000098 | quercetin | NF-kappa-B inhibitor alpha | P25963 | NFKBIA | 4394 | 1 |
| MOL000098 | quercetin | Receptor tyrosine-protein kinase erbB-2 | P04626 | ERBB2 | 4496 | 1 |
| MOL000098 | quercetin | Intercellular adhesion molecule 1 | P05362 | ICAM1 | 4287 | 1 |
| MOL000098 | quercetin | Baculoviral IAP repeat-containing protein 5 | O15392 | BIRC5 | 4044 | 1 |
| MOL000098 | quercetin | G2/mitotic-specific cyclin-B1 | P14635 | CCNB1 | 4219 | 1 |
| MOL000098 | quercetin | Solute carrier family 2, facilitated glucose transporter member 4 | P14672 | SLC2A4 | 4535 | 1 |
| MOL000098 | quercetin | CD40 ligand | P29965 | CD40LG | 4107 | 1 |
| MOL000098 | quercetin | Proto-oncogene c-Fos | P01100 | FOS | 4478 | 1 |
| MOL000098 | quercetin | Eukaryotic translation initiation factor 6 | P56537 | EIF6 | 4197 | 1 |
| MOL000098 | quercetin | Apoptosis regulator BAX | Q07812 | BAX | 4033 | 1 |
| MOL000098 | quercetin | Activator of 90 kDa heat shock protein ATPase homolog 1 | O95433 | AHSA1 | 4007 | 1 |
| MOL000098 | quercetin | Hypoxia-inducible factor 1-alpha | Q16665 | HIF1A | 4263 | 1 |
| MOL000098 | quercetin | Protein CBFA2T1 | Q06455 | RUNX1T1 | 4464 | 1 |
| MOL000098 | quercetin | Plasminogen activator inhibitor 1 | P05121 | SERPINE1 | 4429 | 1 |
| MOL000098 | quercetin | 26S proteasome non-ATPase regulatory subunit 3 | O43242 | PSMD3 | 3993 | 1 |
| MOL000098 | quercetin | ETS domain-containing protein Elk-1 | P19419 | ELK1 | 4194 | 1 |
| MOL000098 | quercetin | Caspase-8 | Q14790 | CASP8 | 4089 | 1 |
| MOL000098 | quercetin | RAF proto-oncogene serine/threonine-protein kinase | P04049 | RAF1 | 4491 | 1 |
| MOL000098 | quercetin | Protein kinase C alpha type | P17252 | PRKCA | 4469 | 1 |
| MOL000098 | quercetin | Signal transducer and activator of transcription 1-alpha/beta | P42224 | STAT1 | 4525 | 1 |
| MOL000098 | quercetin | Caveolin-1 | Q03135 | CAV1 | 4095 | 1 |
| MOL000098 | quercetin | Myc proto-oncogene protein | P01106 | MYC | 4372 | 1 |
| MOL000098 | quercetin | Cytochrome P450 1A1 | P04798 | CYP1A1 | 4149 | 1 |
| MOL000098 | quercetin | Interleukin-8 | P10145 | CXCL8 | 4303 | 1 |
| MOL000098 | quercetin | Protein kinase C beta type | P05771 | PRKCB | 4470 | 1 |
| MOL000098 | quercetin | Dual oxidase 2 | Q9NRD8 | DUOX2 | 4174 | 1 |
| MOL000098 | quercetin | Heat shock protein beta-1 | P04792 | HSPB1 | 4247 | 1 |
| MOL000098 | quercetin | Transforming growth factor beta-1 | O43294 | TGFB1I1 | 4568 | 1 |
| MOL000098 | quercetin | Nuclear receptor subfamily 1 group I member 2 | O75469 | NR1I2 | 4403 | 1 |
| MOL000098 | quercetin | Cytochrome P450 1B1 | Q16678 | CYP1B1 | 4150 | 1 |
| MOL000098 | quercetin | Interleukin-1 alpha | P01583 | IL1A | 4297 | 1 |
| MOL000098 | quercetin | Neutrophil cytosol factor 1 | P14598 | NCF1 | 4391 | 1 |
| MOL000098 | quercetin | ATP-binding cassette sub-family G member 2 | Q9UNQ0 | ABCG2 | 4038 | 1 |
| MOL000098 | quercetin | Hyaluronan synthase 2 | Q92819 | HAS2 | 4262 | 1 |
| MOL000098 | quercetin | Nuclear factor erythroid 2-related factor 2 | Q16236 | NFE2L2 | 4398 | 1 |
| MOL000098 | quercetin | Poly [ADP-ribose] polymerase 1 | P09874 | PARP1 | 4435 | 1 |
| MOL000098 | quercetin | C-X-C motif chemokine 11 | O14625 | CXCL11 | 4137 | 1 |
| MOL000098 | quercetin | C-X-C motif chemokine 2 | P19875 | CXCL2 | 4138 | 1 |
| MOL000098 | quercetin | DDB1- and CUL4-associated factor 5 | Q96JK2 | DCAF5 | 4159 | 1 |
| MOL000098 | quercetin | Nuclear receptor subfamily 1 group I member 3 | Q14994 | NR1I3 | 4404 | 1 |
| MOL000098 | quercetin | Serine/threonine-protein kinase Chk2 | O96017 | CHEK2 | 4513 | 1 |
| MOL000098 | quercetin | Claudin-4 | O14493 | CLDN4 | 4125 | 1 |
| MOL000098 | quercetin | Peroxisome proliferator-activated receptor alpha | Q07869 | PPARA | 4420 | 1 |
| MOL000098 | quercetin | Peroxisome proliferator-activated receptor delta | Q03181 | PPARD | 4421 | 1 |
| MOL000098 | quercetin | Heat shock factor protein 1 | Q00613 | HSF1 | 4246 | 1 |
| MOL000098 | quercetin | C-reactive protein | P02741 | CRP | 4130 | 1 |
| MOL000098 | quercetin | C-X-C motif chemokine 10 | P02778 | CXCL10 | 4136 | 1 |
| MOL000098 | quercetin | Inhibitor of nuclear factor kappa-B kinase subunit alpha | O15111 | CHUK | 4270 | 1 |
| MOL000098 | quercetin | Osteopontin | P10451 | SPP1 | 4411 | 1 |
| MOL000098 | quercetin | Runt-related transcription factor 2 | Q13950 | RUNX2 | 4505 | 1 |
| MOL000098 | quercetin | Ras association domain-containing protein 1 | Q9NS23 | RASSF1 | 4492 | 1 |
| MOL000098 | quercetin | Transcription factor E2F1 | Q01094 | E2F1 | 4560 | 1 |
| MOL000098 | quercetin | Transcription factor E2F2 | Q14209 | E2F2 | 4561 | 1 |
| MOL000098 | quercetin | Insulin-like growth factor-binding protein 3 | P17936 | IGFBP3 | 4276 | 1 |
| MOL000098 | quercetin | Insulin-like growth factor II | P01344 | IGF2 | 4279 | 1 |
| MOL000098 | quercetin | Interferon regulatory factor 1 | P10914 | IRF1 | 4290 | 1 |
| MOL000098 | quercetin | Receptor tyrosine-protein kinase erbB-3 | P21860 | ERBB3 | 4497 | 1 |
| MOL000098 | quercetin | Type I iodothyronine deiodinase | P49895 | DIO1 | 4589 | 1 |
| MOL000098 | quercetin | Procollagen C-endopeptidase enhancer 1 | Q15113 | PCOLCE | 4443 | 1 |
| MOL000098 | quercetin | Puromycin-sensitive aminopeptidase | P55786 | NPEPPS | 4484 | 1 |
| MOL000098 | quercetin | Hexokinase-2 | P52789 | HK2 | 4252 | 1 |
| MOL000098 | quercetin | Homeobox protein Nkx-3.1 | Q99801 | NKX3-1 | 4259 | 1 |
| MOL000098 | quercetin | Ras GTPase-activating protein 1 | P20936 | RASA1 | 4493 | 1 |
| MOL001494 | Mandenol | Prostaglandin G/H synthase 1 | P23219 | PTGS1 | 6 | 20 |
| MOL001494 | Mandenol | Prostaglandin G/H synthase 2 | P35354 | PTGS2 | 94 | 290 |
| MOL001494 | Mandenol | Nuclear receptor coactivator 2 | Q15596 | NCOA2 | 3276 | 6241 |
| MOL008400 | glycitein | Prostaglandin G/H synthase 1 | P23219 | PTGS1 | 6 | 20 |
| MOL008400 | glycitein | Estrogen receptor | P03372 | ESR1 | 46 | 136 |
| MOL008400 | glycitein | Androgen receptor | P10275 | AR | 48 | 146 |
| MOL008400 | glycitein | Peroxisome proliferator activated receptor gamma | P37231 | PPARG | 78 | 238 |
| MOL008400 | glycitein | Prostaglandin G/H synthase 2 | P35354 | PTGS2 | 94 | 290 |
| MOL008400 | glycitein | Retinoic acid receptor RXR-alpha | P19793 | RXRA | 158 | 459 |
| MOL008400 | glycitein | CGMP-inhibited 3',5'-cyclic phosphodiesterase A | Q14432 | PDE3A | 172 | 485 |
| MOL008400 | glycitein | Estrogen receptor beta | Q92731 | ESR2 | 307 | 869 |
| MOL008400 | glycitein | Mitogen-activated protein kinase 14 | Q16539 | MAPK14 | 402 | 1540 |
| MOL008400 | glycitein | Glycogen synthase kinase-3 beta | P49841 | GSK3B | 422 | 1721 |
| MOL008400 | glycitein | Heat shock protein HSP 90 | P07900 | HSP90AA1 | 444 | 1939 |
| MOL008400 | glycitein | Cell division protein kinase 2 | P24941 | CDK2 | 482 | 2240 |
| MOL008400 | glycitein | Serine/threonine-protein kinase Chk1 | O14757 | CHEK1 | 647 | 5790 |
| MOL008400 | glycitein | Trypsin-1 | P07477 | PRSS1 | 2928 | 3176 |
| MOL008400 | glycitein | Proto-oncogene serine/threonine-protein kinase Pim-1 | P11309 | PIM1 | 2966 | 2347 |
| MOL008400 | glycitein | Cyclin-A2 | P20248 | CCNA2 | 3025 | 6235 |
| MOL008400 | glycitein | Calmodulin | P62158 | CALM1 | 3907 | 465 |
| MOL008400 | glycitein | mRNA of PKA Catalytic Subunit C-alpha | P17612 | PRKACA | 699 | 6263 |
| MOL008400 | glycitein | Nuclear receptor coactivator 1 | Q15788 | NCOA1 | 3279 | 6228 |
| MOL008400 | glycitein | Nitric oxide synthase, inducible | P35228 | NOS2 | 3 | 7 |
| MOL008400 | glycitein | Amyloid beta A4 protein | P05067 | APP | 648 | 5800 |
| MOL008400 | glycitein | Collagenase 3 | P45452 | MMP13 | 377 | 1330 |
| MOL008400 | glycitein | Neutrophil collagenase | P22894 | MMP8 | 468 | 2154 |
| MOL005406 | atropine | Dopamine D1 receptor | P21728 | DRD1 | 7 | 23 |
| MOL005406 | atropine | Muscarinic acetylcholine receptor M3 | P20309 | CHRM3 | 16 | 51 |
| MOL005406 | atropine | Muscarinic acetylcholine receptor M1 | P11229 | CHRM1 | 38 | 103 |
| MOL005406 | atropine | Beta-1 adrenergic receptor | P08588 | ADRB1 | 63 | 193 |
| MOL005406 | atropine | Muscarinic acetylcholine receptor M5 | P08912 | CHRM5 | 87 | 274 |
| MOL005406 | atropine | Alpha-2A adrenergic receptor | P08913 | ADRA2A | 105 | 318 |
| MOL005406 | atropine | Alpha-2C adrenergic receptor | P18825 | ADRA2C | 126 | 378 |
| MOL005406 | atropine | Muscarinic acetylcholine receptor M4 | P08173 | CHRM4 | 154 | 450 |
| MOL005406 | atropine | Delta-type opioid receptor | P41143 | OPRD1 | 163 | 467 |
| MOL005406 | atropine | 5-hydroxytryptamine 2A receptor | P28223 | HTR2A | 175 | 502 |
| MOL005406 | atropine | Sodium-dependent noradrenaline transporter | P23975 | SLC6A2 | 186 | 540 |
| MOL005406 | atropine | Alpha-1A adrenergic receptor | P35348 | ADRA1A | 191 | 556 |
| MOL005406 | atropine | 5-hydroxytryptamine 2C receptor | P28335 | HTR2C | 203 | 590 |
| MOL005406 | atropine | Muscarinic acetylcholine receptor M2 | P08172 | CHRM2 | 210 | 617 |
| MOL005406 | atropine | Alpha-2B adrenergic receptor | P18089 | ADRA2B | 214 | 629 |
| MOL005406 | atropine | Alpha-1B adrenergic receptor | P35368 | ADRA1B | 216 | 632 |
| MOL005406 | atropine | Sodium-dependent dopamine transporter | Q01959 | SLC6A3 | 239 | 713 |
| MOL005406 | atropine | Beta-2 adrenergic receptor | P07550 | ADRB2 | 261 | 766 |
| MOL005406 | atropine | Sodium-dependent serotonin transporter | P31645 | SLC6A4 | 290 | 824 |
| MOL005406 | atropine | D(2) dopamine receptor | P14416 | DRD2 | 292 | 831 |
| MOL005406 | atropine | Mu-type opioid receptor | P35372 | OPRM1 | 299 | 847 |
| MOL005406 | atropine | Gamma-aminobutyric acid receptor subunit alpha-1 | P14867 | GABRA1 | 309 | 872 |
| MOL005406 | atropine | 5-hydroxytryptamine 1B receptor | P28222 | HTR1B | 310 | 885 |
| MOL005406 | atropine | Histamine H1 receptor | P35367 | HRH1 | 173 | 492 |
| MOL005406 | atropine | 5-hydroxytryptamine 1A receptor | P08908 | HTR1A | 106 | 320 |
| MOL009646 | 7-O-Methylluteolin-6-C-beta-glucoside_qt | Prostaglandin G/H synthase 2 | P35354 | PTGS2 | 94 | 290 |
| MOL009646 | 7-O-Methylluteolin-6-C-beta-glucoside_qt | DNA topoisomerase II | P11388 | TOP2A | 287 | 817 |
| MOL009646 | 7-O-Methylluteolin-6-C-beta-glucoside_qt | Heat shock protein HSP 90 | P07900 | HSP90AA1 | 444 | 1939 |
| MOL009646 | 7-O-Methylluteolin-6-C-beta-glucoside_qt | Nuclear receptor coactivator 2 | Q15596 | NCOA2 | 3276 | 6241 |
| MOL009646 | 7-O-Methylluteolin-6-C-beta-glucoside_qt | Calmodulin | P62158 | CALM1 | 3907 | 465 |
