# Supplementary material for: Inhibition of Iron Death by Lycium barbarum Polysaccharides Ameliorates Myocardial Injury in Sepsis: A Pharmacological Mechanism Study Based on the NRF2/HO‐1 Pathway
Source: Food Sci Nutr. 2025 Sep 17;13(9):e70835. doi: 10.1002/fsn3.70835 (PMC12441308; doi:10.1002/fsn3.70835)
Supplement: Supplementary file 2 — Table S2: fsn370835‐sup‐0002‐TableS2.docx. [file FSN3-13-e70835-s002.docx]

| Element | CHEA | ChIP_Atlas | ENCODE | FIMO_JASPAR | GTRD | hTFtarget | PWMEnrich_JASPAR |
| --- | --- | --- | --- | --- | --- | --- | --- |
| AFF1 | 0 | 1 | 0 | 0 | 1 | 0 | 0 |
| AFF4 | 0 | 1 | 0 | 0 | 1 | 0 | 0 |
| AGO1 | 0 | 1 | 0 | 0 | 1 | 0 | 0 |
| AGO2 | 0 | 0 | 0 | 0 | 1 | 0 | 0 |
| AHDC1 | 0 | 1 | 0 | 0 | 0 | 0 | 0 |
| AHR | 1 | 1 | 0 | 0 | 1 | 1 | 0 |
| AHRR | 0 | 1 | 0 | 0 | 1 | 0 | 0 |
| AKAP8 | 0 | 1 | 0 | 0 | 0 | 0 | 0 |
| AMH | 0 | 0 | 0 | 0 | 1 | 0 | 0 |
| APC | 0 | 1 | 0 | 0 | 1 | 0 | 0 |
| APOBEC3B | 0 | 0 | 0 | 0 | 1 | 0 | 0 |
| APP | 0 | 0 | 0 | 0 | 1 | 0 | 0 |
| AR | 0 | 1 | 0 | 0 | 1 | 1 | 0 |
| ARHGAP35 | 0 | 1 | 0 | 0 | 0 | 0 | 0 |
| ARID1A | 0 | 1 | 0 | 0 | 1 | 0 | 0 |
| ARID1B | 0 | 1 | 0 | 0 | 1 | 0 | 0 |
| ARID2 | 0 | 1 | 0 | 0 | 1 | 0 | 0 |
| ARID3A | 0 | 0 | 1 | 0 | 1 | 1 | 0 |
| ARID4A | 0 | 1 | 0 | 0 | 0 | 0 | 0 |
| ARID4B | 0 | 1 | 0 | 0 | 1 | 0 | 0 |
| ARID5B | 0 | 1 | 0 | 0 | 0 | 0 | 0 |
| ARNT | 1 | 1 | 0 | 0 | 1 | 1 | 0 |
| ARNTL | 0 | 0 | 0 | 0 | 1 | 0 | 0 |
| ARRB1 | 0 | 0 | 0 | 0 | 1 | 0 | 0 |
| ASCL1 | 0 | 1 | 0 | 1 | 1 | 1 | 0 |
| ASCL2 | 0 | 1 | 0 | 0 | 1 | 1 | 0 |
| ASF1A | 0 | 0 | 0 | 0 | 1 | 0 | 0 |
| ASH2L | 1 | 1 | 0 | 0 | 1 | 0 | 0 |
| ASPSCR1 | 0 | 1 | 0 | 0 | 0 | 0 | 0 |
| ASXL1 | 0 | 1 | 0 | 0 | 0 | 0 | 0 |
| ATAD3A | 0 | 1 | 0 | 0 | 0 | 0 | 0 |
| ATF1 | 0 | 1 | 1 | 0 | 1 | 0 | 0 |
| ATF2 | 0 | 1 | 1 | 0 | 1 | 0 | 0 |
| ATF3 | 1 | 1 | 1 | 0 | 1 | 1 | 0 |
| ATF4 | 0 | 1 | 0 | 0 | 1 | 0 | 0 |
| ATF7 | 0 | 1 | 0 | 0 | 1 | 0 | 0 |
| ATF7IP | 0 | 0 | 0 | 0 | 1 | 0 | 0 |
| ATRX | 0 | 1 | 0 | 0 | 1 | 1 | 0 |
| BACH1 | 0 | 1 | 1 | 0 | 1 | 1 | 0 |
| BACH2 | 0 | 1 | 0 | 0 | 1 | 1 | 0 |
| BAHCC1 | 0 | 1 | 0 | 0 | 0 | 0 | 0 |
| BANF1 | 0 | 1 | 0 | 0 | 0 | 0 | 0 |
| BANP | 0 | 1 | 0 | 0 | 0 | 0 | 0 |
| BAP1 | 0 | 1 | 0 | 0 | 1 | 0 | 0 |
| BARHL1 | 0 | 0 | 0 | 0 | 1 | 1 | 0 |
| BATF | 0 | 1 | 1 | 0 | 1 | 0 | 0 |
| BATF3 | 0 | 1 | 0 | 0 | 1 | 0 | 0 |
| BCHE | 0 | 0 | 0 | 0 | 1 | 0 | 0 |
| BCL11A | 0 | 1 | 1 | 0 | 1 | 0 | 0 |
| BCL11B | 0 | 1 | 0 | 0 | 1 | 0 | 0 |
| BCL3 | 0 | 1 | 1 | 0 | 1 | 1 | 0 |
| BCL6 | 0 | 1 | 0 | 0 | 1 | 1 | 0 |
| BCL6B | 0 | 0 | 0 | 0 | 0 | 0 | 1 |
| BCLAF1 | 0 | 0 | 1 | 0 | 1 | 0 | 0 |
| BCOR | 0 | 1 | 0 | 0 | 0 | 1 | 0 |
| BCORL1 | 0 | 1 | 0 | 0 | 0 | 0 | 0 |
| BHLHE40 | 0 | 1 | 1 | 0 | 1 | 1 | 0 |
| BICRA | 0 | 0 | 0 | 0 | 1 | 0 | 0 |
| BMI1 | 0 | 1 | 0 | 0 | 1 | 1 | 0 |
| BPTF | 0 | 0 | 0 | 0 | 1 | 0 | 0 |
| BRCA1 | 0 | 1 | 1 | 0 | 1 | 1 | 0 |
| BRD1 | 0 | 0 | 0 | 0 | 1 | 0 | 0 |
| BRD2 | 0 | 1 | 0 | 0 | 1 | 1 | 0 |
| BRD3 | 0 | 1 | 0 | 0 | 1 | 1 | 0 |
| BRD4 | 0 | 1 | 0 | 0 | 1 | 1 | 0 |
| BRD7 | 0 | 1 | 0 | 0 | 1 | 0 | 0 |
| BRD8 | 0 | 1 | 0 | 0 | 0 | 0 | 0 |
| BRD9 | 0 | 1 | 0 | 0 | 1 | 0 | 0 |
| BRPF3 | 0 | 1 | 0 | 0 | 1 | 0 | 0 |
| c-myc | 0 | 0 | 0 | 0 | 1 | 0 | 0 |
| C17orf49 | 0 | 1 | 0 | 0 | 0 | 0 | 0 |
| CASZ1 | 0 | 1 | 0 | 0 | 0 | 0 | 0 |
| CBFA2T2 | 0 | 1 | 0 | 0 | 1 | 0 | 0 |
| CBFA2T3 | 0 | 1 | 0 | 0 | 1 | 0 | 0 |
| CBFB | 0 | 1 | 0 | 0 | 1 | 1 | 0 |
| CBX1 | 0 | 1 | 0 | 0 | 1 | 0 | 0 |
| CBX2 | 0 | 0 | 0 | 0 | 1 | 0 | 0 |
| CBX3 | 0 | 1 | 1 | 0 | 1 | 1 | 0 |
| CBX5 | 0 | 1 | 0 | 0 | 1 | 0 | 0 |
| CBX6 | 0 | 0 | 0 | 0 | 1 | 0 | 0 |
| CBX8 | 0 | 0 | 0 | 0 | 1 | 1 | 0 |
| CC2D1A | 0 | 1 | 0 | 0 | 1 | 0 | 0 |
| CCAR2 | 0 | 0 | 0 | 0 | 1 | 0 | 0 |
| CCND1 | 1 | 0 | 0 | 0 | 0 | 0 | 0 |
| CCND2 | 0 | 1 | 0 | 0 | 1 | 0 | 0 |
| CCNT1 | 0 | 1 | 0 | 0 | 0 | 0 | 0 |
| CCNT2 | 0 | 1 | 1 | 0 | 1 | 1 | 0 |
| CDC73 | 0 | 0 | 0 | 0 | 1 | 0 | 0 |
| CDK12 | 0 | 0 | 0 | 0 | 1 | 0 | 0 |
| CDK2 | 0 | 0 | 0 | 0 | 1 | 0 | 0 |
| CDK6 | 0 | 1 | 0 | 0 | 0 | 0 | 0 |
| CDK7 | 0 | 1 | 0 | 0 | 1 | 1 | 0 |
| CDK8 | 0 | 1 | 0 | 0 | 1 | 1 | 0 |
| CDK9 | 0 | 1 | 0 | 0 | 1 | 1 | 0 |
| CDKN1B | 0 | 1 | 0 | 0 | 1 | 0 | 0 |
| CDX1 | 0 | 0 | 0 | 1 | 0 | 0 | 0 |
| CDX2 | 1 | 0 | 0 | 1 | 1 | 1 | 0 |
| CDX4 | 0 | 0 | 0 | 1 | 0 | 0 | 0 |
| CDYL2 | 0 | 1 | 0 | 0 | 0 | 0 | 0 |
| CEBPA | 0 | 1 | 0 | 0 | 1 | 1 | 0 |
| CEBPB | 0 | 1 | 1 | 0 | 1 | 1 | 0 |
| CEBPD | 1 | 1 | 1 | 0 | 1 | 1 | 0 |
| CEBPG | 0 | 1 | 0 | 0 | 1 | 0 | 0 |
| CEBPZ | 0 | 0 | 1 | 0 | 0 | 0 | 0 |
| CENPA | 0 | 0 | 0 | 0 | 1 | 0 | 0 |
| CHAF1B | 0 | 0 | 0 | 0 | 1 | 0 | 0 |
| CHAMP1 | 0 | 1 | 0 | 0 | 0 | 0 | 0 |
| CHD1 | 0 | 1 | 1 | 0 | 1 | 1 | 0 |
| CHD2 | 0 | 1 | 1 | 0 | 1 | 0 | 0 |
| CHD4 | 0 | 1 | 1 | 0 | 1 | 0 | 0 |
| CHD6 | 0 | 1 | 0 | 0 | 0 | 0 | 0 |
| CHD7 | 0 | 0 | 1 | 0 | 1 | 0 | 0 |
| CHD8 | 0 | 1 | 0 | 0 | 1 | 0 | 0 |
| CIC | 0 | 1 | 0 | 0 | 0 | 0 | 0 |
| CLOCK | 0 | 0 | 0 | 0 | 1 | 0 | 0 |
| CNOT3 | 0 | 1 | 0 | 0 | 1 | 0 | 0 |
| CPSF2 | 0 | 1 | 0 | 0 | 0 | 0 | 0 |
| CPSF3 | 0 | 1 | 0 | 0 | 0 | 0 | 0 |
| CPSF4 | 0 | 1 | 0 | 0 | 0 | 0 | 0 |
| CREB1 | 0 | 1 | 1 | 0 | 1 | 1 | 0 |
| CREB3 | 0 | 0 | 0 | 0 | 1 | 0 | 0 |
| CREB3L1 | 0 | 0 | 0 | 0 | 1 | 0 | 0 |
| CREB3L4 | 0 | 0 | 0 | 0 | 1 | 0 | 0 |
| CREB5 | 0 | 0 | 0 | 0 | 1 | 0 | 0 |
| CREBBP | 0 | 1 | 1 | 0 | 1 | 1 | 0 |
| CREM | 0 | 1 | 0 | 0 | 1 | 0 | 0 |
| CRTC2 | 0 | 1 | 0 | 0 | 1 | 0 | 0 |
| CSNK2A1 | 0 | 0 | 0 | 0 | 1 | 0 | 0 |
| CSTF2 | 0 | 1 | 0 | 0 | 0 | 0 | 0 |
| CTBP1 | 0 | 1 | 0 | 0 | 1 | 0 | 0 |
| CTBP2 | 0 | 1 | 1 | 0 | 1 | 1 | 0 |
| CTCF | 0 | 1 | 1 | 1 | 1 | 1 | 1 |
| CTCFL | 0 | 1 | 1 | 1 | 1 | 1 | 0 |
| CTNNB1 | 1 | 1 | 0 | 0 | 1 | 1 | 0 |
| CUX1 | 1 | 1 | 1 | 0 | 0 | 0 | 0 |
| CXXC1 | 0 | 0 | 0 | 0 | 1 | 0 | 0 |
| CXXC4 | 0 | 0 | 0 | 0 | 1 | 0 | 0 |
| CXXC5 | 0 | 1 | 0 | 0 | 0 | 0 | 0 |
| DACH1 | 0 | 1 | 0 | 0 | 1 | 0 | 0 |
| DAXX | 0 | 1 | 0 | 0 | 1 | 0 | 0 |
| DBP | 0 | 0 | 0 | 0 | 1 | 0 | 0 |
| DCP1A | 0 | 0 | 0 | 0 | 1 | 0 | 0 |
| DCP2 | 0 | 1 | 0 | 0 | 0 | 0 | 0 |
| DDIT3 | 0 | 1 | 0 | 0 | 1 | 0 | 0 |
| DDX21 | 0 | 1 | 0 | 0 | 1 | 0 | 0 |
| DDX5 | 0 | 0 | 0 | 0 | 1 | 1 | 0 |
| DEAF1 | 0 | 1 | 0 | 0 | 1 | 0 | 0 |
| DEK | 0 | 0 | 0 | 0 | 1 | 0 | 0 |
| DIDO1 | 0 | 1 | 0 | 0 | 0 | 0 | 0 |
| DLX1 | 0 | 0 | 0 | 0 | 1 | 0 | 0 |
| DLX5 | 0 | 1 | 0 | 0 | 0 | 0 | 0 |
| DLX6 | 0 | 1 | 0 | 0 | 0 | 0 | 0 |
| DMAP1 | 0 | 1 | 0 | 0 | 1 | 0 | 0 |
| DMC1 | 0 | 0 | 0 | 0 | 1 | 0 | 0 |
| DNMT3B | 0 | 0 | 0 | 0 | 1 | 0 | 0 |
| DOT1L | 0 | 1 | 0 | 0 | 1 | 0 | 0 |
| DPF2 | 0 | 1 | 0 | 0 | 1 | 0 | 0 |
| DRAP1 | 0 | 1 | 0 | 0 | 1 | 0 | 0 |
| DUX4 | 0 | 0 | 0 | 1 | 1 | 1 | 1 |
| DYRK1A | 0 | 1 | 0 | 0 | 0 | 0 | 0 |
| DZIP1 | 0 | 1 | 0 | 0 | 0 | 0 | 0 |
| E2F1 | 1 | 1 | 1 | 0 | 1 | 1 | 0 |
| E2F3 | 0 | 1 | 0 | 0 | 1 | 1 | 0 |
| E2F4 | 1 | 1 | 1 | 0 | 1 | 1 | 0 |
| E2F5 | 0 | 1 | 0 | 0 | 1 | 0 | 0 |
| E2F6 | 0 | 1 | 1 | 1 | 1 | 1 | 0 |
| E2F7 | 0 | 0 | 0 | 0 | 1 | 1 | 0 |
| E2F8 | 0 | 0 | 0 | 0 | 1 | 1 | 0 |
| E4F1 | 0 | 0 | 0 | 0 | 1 | 0 | 0 |
| EBF1 | 0 | 1 | 1 | 0 | 1 | 1 | 0 |
| EBF3 | 0 | 0 | 0 | 0 | 1 | 0 | 0 |
| EBP | 0 | 0 | 0 | 0 | 1 | 0 | 0 |
| EEA1 | 0 | 1 | 0 | 0 | 0 | 0 | 0 |
| EED | 0 | 1 | 0 | 0 | 1 | 0 | 0 |
| EGR1 | 1 | 1 | 1 | 1 | 1 | 1 | 0 |
| EGR2 | 0 | 1 | 0 | 1 | 1 | 0 | 0 |
| EGR3 | 0 | 0 | 0 | 1 | 1 | 0 | 0 |
| EGR4 | 0 | 0 | 0 | 1 | 0 | 0 | 1 |
| EHF | 0 | 0 | 0 | 1 | 1 | 0 | 0 |
| EHMT2 | 0 | 1 | 0 | 0 | 1 | 0 | 0 |
| ELF1 | 0 | 1 | 1 | 1 | 1 | 1 | 0 |
| ELF3 | 0 | 1 | 0 | 1 | 1 | 0 | 0 |
| ELF5 | 1 | 0 | 0 | 0 | 1 | 1 | 0 |
| ELK1 | 0 | 0 | 1 | 0 | 0 | 1 | 0 |
| ELK3 | 0 | 0 | 0 | 0 | 1 | 1 | 0 |
| ELK4 | 0 | 0 | 0 | 0 | 1 | 0 | 0 |
| ELL2 | 0 | 1 | 0 | 0 | 1 | 1 | 0 |
| EMSY | 0 | 1 | 0 | 0 | 1 | 0 | 0 |
| EN1 | 0 | 0 | 0 | 0 | 1 | 0 | 0 |
| EOMES | 1 | 0 | 0 | 0 | 0 | 1 | 0 |
| EP300 | 1 | 1 | 1 | 0 | 1 | 1 | 0 |
| EP400 | 0 | 1 | 0 | 0 | 1 | 0 | 0 |
| EPAS1 | 0 | 1 | 0 | 0 | 1 | 0 | 0 |
| ERCC3 | 0 | 1 | 0 | 0 | 1 | 0 | 0 |
| ERCC6 | 0 | 0 | 0 | 0 | 1 | 0 | 0 |
| ERF | 0 | 1 | 0 | 0 | 1 | 0 | 0 |
| ERG | 0 | 1 | 0 | 0 | 1 | 1 | 0 |
| ESCO2 | 0 | 0 | 0 | 0 | 1 | 0 | 0 |
| ESR1 | 1 | 1 | 0 | 0 | 1 | 1 | 0 |
| ESR2 | 0 | 0 | 0 | 1 | 1 | 0 | 0 |
| ESRRA | 0 | 0 | 0 | 1 | 1 | 1 | 0 |
| ESRRB | 1 | 0 | 0 | 0 | 0 | 0 | 0 |
| ETS1 | 0 | 1 | 1 | 0 | 1 | 1 | 0 |
| ETS2 | 0 | 0 | 0 | 0 | 0 | 1 | 0 |
| ETV1 | 0 | 1 | 0 | 1 | 1 | 1 | 0 |
| ETV2 | 0 | 0 | 0 | 0 | 1 | 0 | 0 |
| ETV2::FIGLA | 0 | 0 | 0 | 1 | 0 | 0 | 0 |
| ETV2::FOXI1 | 0 | 0 | 0 | 1 | 0 | 0 | 0 |
| ETV3 | 0 | 1 | 0 | 0 | 0 | 0 | 0 |
| ETV4 | 0 | 1 | 0 | 0 | 1 | 0 | 0 |
| ETV5 | 0 | 1 | 0 | 0 | 1 | 0 | 0 |
| ETV5::FIGLA | 0 | 0 | 0 | 1 | 0 | 0 | 0 |
| ETV5::FOXO1 | 0 | 0 | 0 | 1 | 0 | 0 | 0 |
| ETV6 | 0 | 1 | 0 | 0 | 1 | 0 | 0 |
| ETV7 | 0 | 0 | 0 | 0 | 1 | 0 | 0 |
| EZH1 | 0 | 0 | 0 | 0 | 0 | 1 | 0 |
| EZH2 | 0 | 1 | 1 | 0 | 1 | 0 | 0 |
| FEV | 0 | 0 | 0 | 0 | 1 | 0 | 0 |
| FEZF1 | 0 | 1 | 0 | 0 | 1 | 0 | 0 |
| FGFR1 | 0 | 0 | 0 | 0 | 1 | 0 | 0 |
| FIP1L1 | 0 | 1 | 0 | 0 | 1 | 0 | 0 |
| FLI1 | 1 | 1 | 1 | 0 | 1 | 1 | 0 |
| FOS | 0 | 1 | 1 | 0 | 1 | 1 | 0 |
| FOSL1 | 0 | 1 | 1 | 0 | 1 | 0 | 0 |
| FOSL2 | 0 | 1 | 1 | 0 | 1 | 0 | 0 |
| FOXA1 | 0 | 1 | 1 | 1 | 1 | 1 | 0 |
| FOXA2 | 1 | 1 | 1 | 1 | 1 | 1 | 1 |
| FOXA3 | 0 | 1 | 0 | 1 | 1 | 0 | 1 |
| FOXB1 | 0 | 0 | 0 | 1 | 0 | 0 | 0 |
| FOXC1 | 0 | 1 | 0 | 1 | 0 | 0 | 0 |
| FOXC2 | 0 | 0 | 0 | 1 | 0 | 0 | 0 |
| FOXD1 | 0 | 0 | 0 | 1 | 0 | 0 | 1 |
| FOXD2 | 0 | 0 | 0 | 1 | 1 | 1 | 0 |
| FOXE1 | 0 | 0 | 0 | 1 | 0 | 0 | 0 |
| FOXG1 | 0 | 0 | 0 | 1 | 0 | 0 | 1 |
| FOXH1 | 0 | 0 | 0 | 1 | 1 | 1 | 0 |
| FOXI1 | 0 | 0 | 0 | 1 | 0 | 0 | 0 |
| FOXJ3 | 0 | 1 | 0 | 0 | 0 | 0 | 0 |
| FOXK1 | 0 | 1 | 0 | 1 | 0 | 0 | 0 |
| FOXK2 | 0 | 1 | 0 | 1 | 1 | 0 | 0 |
| FOXL1 | 0 | 0 | 0 | 1 | 0 | 0 | 0 |
| FOXL2 | 0 | 1 | 0 | 0 | 0 | 0 | 0 |
| FOXM1 | 0 | 1 | 1 | 0 | 1 | 1 | 0 |
| FOXN3 | 0 | 0 | 0 | 1 | 0 | 0 | 1 |
| FOXO1 | 0 | 1 | 0 | 0 | 1 | 0 | 0 |
| FOXO3 | 0 | 0 | 0 | 0 | 1 | 1 | 0 |
| FOXO4 | 0 | 0 | 0 | 1 | 0 | 0 | 0 |
| FOXO6 | 0 | 0 | 0 | 1 | 0 | 0 | 0 |
| FOXP1 | 1 | 1 | 0 | 1 | 1 | 1 | 0 |
| FOXP2 | 0 | 1 | 1 | 1 | 1 | 0 | 1 |
| FOXP3 | 0 | 0 | 0 | 1 | 1 | 1 | 1 |
| FOXP4 | 0 | 1 | 0 | 1 | 0 | 0 | 1 |
| FOXS1 | 0 | 0 | 0 | 1 | 0 | 0 | 1 |
| FUBP1 | 0 | 1 | 0 | 0 | 0 | 0 | 0 |
| FUBP3 | 0 | 1 | 0 | 0 | 0 | 0 | 0 |
| FUS | 0 | 1 | 0 | 0 | 1 | 0 | 0 |
| FXR2 | 0 | 1 | 0 | 0 | 1 | 0 | 0 |
| GABPA | 0 | 1 | 1 | 1 | 1 | 1 | 0 |
| GABPB1 | 0 | 1 | 0 | 0 | 1 | 0 | 0 |
| GATA1 | 0 | 1 | 1 | 0 | 1 | 1 | 0 |
| GATA2 | 0 | 1 | 1 | 0 | 1 | 1 | 0 |
| GATA3 | 0 | 1 | 1 | 0 | 1 | 1 | 0 |
| GATA4 | 0 | 1 | 0 | 0 | 1 | 1 | 0 |
| GATA6 | 0 | 1 | 0 | 0 | 1 | 1 | 0 |
| GATAD1 | 0 | 1 | 0 | 0 | 1 | 0 | 0 |
| GATAD2A | 0 | 0 | 0 | 0 | 1 | 0 | 0 |
| GATAD2B | 0 | 1 | 0 | 0 | 1 | 0 | 0 |
| GBX2 | 1 | 0 | 0 | 0 | 0 | 0 | 0 |
| GCM1 | 0 | 0 | 0 | 1 | 0 | 0 | 0 |
| GFI1 | 0 | 1 | 0 | 0 | 1 | 0 | 0 |
| GFI1B | 0 | 0 | 0 | 0 | 1 | 1 | 0 |
| GLI2 | 0 | 0 | 0 | 0 | 1 | 0 | 0 |
| GLI3 | 0 | 0 | 0 | 0 | 1 | 0 | 0 |
| GLI4 | 0 | 0 | 0 | 0 | 1 | 0 | 0 |
| GLIS1 | 0 | 1 | 0 | 0 | 1 | 0 | 0 |
| GLIS2 | 0 | 1 | 0 | 0 | 0 | 0 | 0 |
| GLIS3 | 0 | 0 | 0 | 0 | 1 | 0 | 0 |
| GLMP | 0 | 1 | 0 | 0 | 0 | 0 | 0 |
| GMEB1 | 0 | 1 | 0 | 0 | 0 | 0 | 0 |
| GMEB2 | 0 | 0 | 0 | 0 | 1 | 1 | 0 |
| GPN1 | 0 | 1 | 0 | 0 | 0 | 0 | 0 |
| GRHL1 | 0 | 1 | 0 | 0 | 1 | 0 | 0 |
| GRHL2 | 0 | 1 | 0 | 1 | 1 | 0 | 0 |
| GRHL3 | 0 | 1 | 0 | 0 | 1 | 0 | 0 |
| GTF2B | 0 | 0 | 1 | 0 | 0 | 1 | 0 |
| GTF2E2 | 0 | 1 | 0 | 0 | 0 | 0 | 0 |
| GTF2F1 | 0 | 1 | 1 | 0 | 1 | 0 | 0 |
| GTF2I | 0 | 0 | 0 | 0 | 0 | 1 | 0 |
| GTF3C2 | 0 | 0 | 0 | 0 | 1 | 1 | 0 |
| GTF3C5 | 0 | 0 | 0 | 0 | 1 | 0 | 0 |
| GZF1 | 0 | 1 | 0 | 0 | 0 | 0 | 0 |
| H2AFZ | 0 | 0 | 1 | 0 | 1 | 0 | 0 |
| HAND2 | 0 | 1 | 0 | 0 | 1 | 0 | 0 |
| HBP1 | 0 | 1 | 0 | 0 | 1 | 0 | 0 |
| HCFC1 | 0 | 1 | 1 | 0 | 1 | 1 | 0 |
| HDAC1 | 0 | 1 | 1 | 0 | 1 | 1 | 0 |
| HDAC2 | 0 | 1 | 1 | 0 | 1 | 1 | 0 |
| HDAC3 | 0 | 0 | 0 | 0 | 1 | 1 | 0 |
| HDAC6 | 0 | 0 | 0 | 0 | 1 | 0 | 0 |
| HDGFL2 | 0 | 0 | 0 | 0 | 1 | 0 | 0 |
| HDGFL3 | 0 | 0 | 0 | 0 | 1 | 0 | 0 |
| HES1 | 0 | 0 | 0 | 0 | 1 | 0 | 0 |
| HES2 | 0 | 1 | 0 | 0 | 1 | 0 | 0 |
| HES4 | 0 | 1 | 0 | 0 | 0 | 0 | 0 |
| HEXIM1 | 0 | 1 | 0 | 0 | 1 | 0 | 0 |
| HEY1 | 0 | 0 | 0 | 0 | 1 | 1 | 0 |
| HHEX | 0 | 1 | 0 | 0 | 1 | 0 | 0 |
| HIC1 | 0 | 1 | 0 | 0 | 1 | 0 | 0 |
| HIC2 | 0 | 1 | 0 | 0 | 0 | 0 | 0 |
| HIF1A | 0 | 1 | 0 | 0 | 1 | 0 | 0 |
| HIF3A | 0 | 0 | 0 | 0 | 1 | 0 | 0 |
| HINFP | 0 | 0 | 0 | 1 | 1 | 0 | 0 |
| HIRA | 0 | 0 | 0 | 0 | 1 | 0 | 0 |
| HIVEP1 | 0 | 1 | 0 | 0 | 0 | 0 | 0 |
| HLF | 0 | 1 | 0 | 0 | 1 | 0 | 0 |
| HMBOX1 | 0 | 1 | 0 | 0 | 1 | 0 | 0 |
| HMG20A | 0 | 1 | 0 | 0 | 1 | 0 | 0 |
| HMG20B | 0 | 1 | 0 | 0 | 1 | 0 | 0 |
| HMGA1 | 0 | 0 | 0 | 0 | 1 | 0 | 0 |
| HMGB1 | 0 | 1 | 0 | 0 | 1 | 0 | 0 |
| HMGB2 | 0 | 1 | 0 | 0 | 0 | 0 | 0 |
| HMGN1 | 0 | 1 | 0 | 0 | 0 | 0 | 0 |
| HMGN3 | 0 | 1 | 1 | 0 | 1 | 0 | 0 |
| HMGXB3 | 0 | 1 | 0 | 0 | 0 | 0 | 0 |
| HMGXB4 | 0 | 1 | 0 | 0 | 1 | 0 | 0 |
| HNF1A | 0 | 1 | 0 | 0 | 1 | 0 | 0 |
| HNF1B | 0 | 1 | 0 | 0 | 1 | 0 | 0 |
| HNF4A | 1 | 1 | 1 | 1 | 1 | 1 | 0 |
| HNF4G | 0 | 1 | 1 | 1 | 1 | 1 | 0 |
| HNRNPC | 0 | 1 | 0 | 0 | 1 | 0 | 0 |
| HNRNPH1 | 0 | 0 | 0 | 0 | 1 | 0 | 0 |
| HNRNPK | 0 | 1 | 0 | 0 | 1 | 0 | 0 |
| HNRNPL | 0 | 1 | 0 | 0 | 1 | 0 | 0 |
| HNRNPLL | 0 | 1 | 0 | 0 | 1 | 0 | 0 |
| HNRNPUL1 | 0 | 0 | 0 | 0 | 1 | 0 | 0 |
| HOMEZ | 0 | 0 | 0 | 0 | 1 | 0 | 0 |
| HOXA1 | 0 | 0 | 0 | 0 | 0 | 1 | 0 |
| HOXA2 | 0 | 0 | 0 | 0 | 1 | 0 | 0 |
| HOXA3 | 0 | 1 | 0 | 0 | 0 | 0 | 0 |
| HOXA4 | 0 | 1 | 0 | 0 | 0 | 0 | 0 |
| HOXA5 | 0 | 1 | 0 | 0 | 1 | 0 | 0 |
| HOXA9 | 0 | 0 | 0 | 0 | 1 | 0 | 0 |
| HOXB13 | 0 | 1 | 0 | 0 | 1 | 0 | 0 |
| HOXB4 | 0 | 0 | 0 | 0 | 1 | 0 | 0 |
| HOXB8 | 0 | 0 | 0 | 0 | 1 | 0 | 0 |
| HOXC5 | 0 | 0 | 0 | 0 | 1 | 0 | 0 |
| HOXD1 | 0 | 1 | 0 | 0 | 0 | 0 | 0 |
| HOXD8 | 0 | 0 | 0 | 1 | 0 | 0 | 0 |
| HOXD9 | 0 | 0 | 0 | 1 | 0 | 0 | 0 |
| HSF1 | 0 | 1 | 0 | 0 | 1 | 1 | 0 |
| ID1 | 0 | 0 | 0 | 0 | 1 | 0 | 0 |
| ID3 | 0 | 1 | 0 | 0 | 1 | 0 | 0 |
| IKZF1 | 0 | 1 | 0 | 0 | 1 | 0 | 0 |
| IKZF2 | 0 | 1 | 0 | 0 | 0 | 0 | 0 |
| IKZF3 | 0 | 1 | 0 | 0 | 1 | 0 | 0 |
| IKZF4 | 0 | 1 | 0 | 0 | 0 | 0 | 0 |
| IKZF5 | 0 | 1 | 0 | 0 | 1 | 0 | 0 |
| ILF3 | 0 | 1 | 0 | 0 | 0 | 0 | 0 |
| ING2 | 0 | 1 | 0 | 0 | 0 | 0 | 0 |
| ING5 | 0 | 1 | 0 | 0 | 0 | 0 | 0 |
| INO80 | 0 | 0 | 0 | 0 | 1 | 0 | 0 |
| INSM1 | 0 | 0 | 0 | 1 | 0 | 0 | 0 |
| INSM2 | 0 | 1 | 0 | 0 | 1 | 0 | 0 |
| INSR | 0 | 0 | 0 | 0 | 1 | 0 | 0 |
| INTS10 | 0 | 1 | 0 | 0 | 0 | 0 | 0 |
| INTS11 | 0 | 0 | 0 | 0 | 1 | 0 | 0 |
| INTS12 | 0 | 1 | 0 | 0 | 1 | 0 | 0 |
| INTS13 | 0 | 1 | 0 | 0 | 1 | 0 | 0 |
| INTS3 | 0 | 1 | 0 | 0 | 0 | 0 | 0 |
| INTS6 | 0 | 1 | 0 | 0 | 0 | 0 | 0 |
| IRF1 | 0 | 1 | 1 | 0 | 1 | 1 | 0 |
| IRF2 | 0 | 1 | 0 | 0 | 1 | 0 | 0 |
| IRF3 | 0 | 0 | 1 | 0 | 0 | 0 | 0 |
| IRF4 | 0 | 1 | 1 | 0 | 1 | 0 | 0 |
| IRF9 | 0 | 1 | 0 | 0 | 1 | 0 | 0 |
| ISL1 | 0 | 0 | 0 | 0 | 1 | 0 | 0 |
| ISL2 | 0 | 1 | 0 | 0 | 0 | 0 | 0 |
| ISX | 0 | 1 | 0 | 0 | 0 | 0 | 0 |
| IVNS1ABP | 0 | 0 | 0 | 0 | 1 | 0 | 0 |
| JADE3 | 0 | 1 | 0 | 0 | 0 | 0 | 0 |
| JARID2 | 0 | 0 | 0 | 0 | 1 | 0 | 0 |
| JDP2 | 0 | 1 | 0 | 0 | 1 | 0 | 0 |
| JMJD1C | 0 | 1 | 0 | 0 | 0 | 0 | 0 |
| JMJD6 | 0 | 1 | 0 | 0 | 1 | 1 | 0 |
| JRK | 0 | 1 | 0 | 0 | 0 | 0 | 0 |
| JUN | 1 | 1 | 1 | 0 | 1 | 1 | 0 |
| JUNB | 0 | 1 | 0 | 0 | 1 | 0 | 0 |
| JUND | 0 | 1 | 1 | 0 | 1 | 1 | 0 |
| KAT2A | 0 | 0 | 1 | 0 | 1 | 0 | 0 |
| KAT2B | 0 | 0 | 0 | 0 | 1 | 1 | 0 |
| KAT5 | 0 | 0 | 0 | 0 | 0 | 1 | 0 |
| KAT6A | 0 | 1 | 0 | 0 | 0 | 0 | 0 |
| KAT6B | 0 | 1 | 0 | 0 | 0 | 0 | 0 |
| KAT7 | 0 | 1 | 0 | 0 | 1 | 0 | 0 |
| KAT8 | 0 | 0 | 0 | 0 | 1 | 0 | 0 |
| KDM1A | 0 | 1 | 1 | 0 | 1 | 1 | 0 |
| KDM2A | 0 | 1 | 0 | 0 | 0 | 0 | 0 |
| KDM2B | 0 | 1 | 0 | 0 | 1 | 0 | 0 |
| KDM3A | 0 | 1 | 0 | 0 | 1 | 0 | 0 |
| KDM3B | 0 | 1 | 0 | 0 | 1 | 0 | 0 |
| KDM4A | 0 | 1 | 1 | 0 | 1 | 1 | 0 |
| KDM4B | 0 | 0 | 0 | 0 | 1 | 0 | 0 |
| KDM4C | 0 | 0 | 0 | 0 | 1 | 1 | 0 |
| KDM5A | 0 | 0 | 1 | 0 | 1 | 0 | 0 |
| KDM5B | 0 | 1 | 1 | 0 | 1 | 1 | 0 |
| KDM5C | 0 | 0 | 0 | 0 | 1 | 0 | 0 |
| KDM5D | 0 | 0 | 0 | 0 | 1 | 0 | 0 |
| KDM6A | 0 | 1 | 0 | 0 | 1 | 0 | 0 |
| KDM6B | 0 | 0 | 0 | 0 | 1 | 0 | 0 |
| KDM7A | 0 | 0 | 0 | 0 | 1 | 0 | 0 |
| KLF1 | 0 | 1 | 0 | 1 | 1 | 1 | 0 |
| KLF10 | 0 | 1 | 0 | 1 | 1 | 0 | 0 |
| KLF11 | 0 | 1 | 0 | 1 | 1 | 0 | 0 |
| KLF12 | 0 | 1 | 0 | 1 | 1 | 0 | 0 |
| KLF14 | 0 | 1 | 0 | 1 | 0 | 0 | 0 |
| KLF15 | 0 | 0 | 0 | 1 | 1 | 0 | 0 |
| KLF16 | 0 | 1 | 0 | 1 | 1 | 0 | 0 |
| KLF17 | 0 | 1 | 0 | 1 | 0 | 0 | 0 |
| KLF2 | 0 | 0 | 0 | 1 | 0 | 0 | 0 |
| KLF3 | 0 | 1 | 0 | 1 | 1 | 0 | 0 |
| KLF4 | 1 | 1 | 0 | 1 | 1 | 1 | 0 |
| KLF5 | 0 | 1 | 0 | 0 | 1 | 1 | 0 |
| KLF6 | 0 | 1 | 0 | 0 | 1 | 0 | 0 |
| KLF7 | 0 | 1 | 0 | 1 | 0 | 0 | 0 |
| KLF8 | 0 | 1 | 0 | 0 | 0 | 0 | 0 |
| KLF9 | 0 | 1 | 0 | 0 | 1 | 1 | 0 |
| KMT2A | 0 | 1 | 0 | 0 | 1 | 1 | 0 |
| KMT2B | 0 | 1 | 0 | 0 | 1 | 0 | 0 |
| KMT2C | 0 | 1 | 0 | 0 | 1 | 0 | 0 |
| KMT2D | 0 | 1 | 0 | 0 | 1 | 0 | 0 |
| L3MBTL2 | 0 | 1 | 0 | 0 | 1 | 0 | 0 |
| L3MBTL4 | 0 | 0 | 0 | 0 | 1 | 0 | 0 |
| LARP7 | 0 | 1 | 0 | 0 | 1 | 0 | 0 |
| LBX2 | 0 | 1 | 0 | 0 | 0 | 0 | 0 |
| LCOR | 0 | 1 | 0 | 0 | 0 | 0 | 0 |
| LCORL | 0 | 1 | 0 | 0 | 0 | 0 | 0 |
| LDB1 | 0 | 1 | 0 | 0 | 1 | 0 | 0 |
| LDB2 | 0 | 1 | 0 | 0 | 0 | 0 | 0 |
| LEF1 | 0 | 1 | 0 | 0 | 0 | 0 | 0 |
| LEO1 | 0 | 0 | 0 | 0 | 1 | 0 | 0 |
| LIN54 | 0 | 1 | 0 | 0 | 0 | 0 | 0 |
| LIN9 | 0 | 1 | 0 | 0 | 0 | 0 | 0 |
| LMNA | 0 | 1 | 0 | 0 | 1 | 0 | 0 |
| LMNB1 | 0 | 0 | 0 | 0 | 0 | 1 | 0 |
| LMO1 | 0 | 0 | 0 | 0 | 1 | 0 | 0 |
| LMO2 | 0 | 0 | 0 | 0 | 0 | 1 | 0 |
| LMO3 | 0 | 1 | 0 | 0 | 0 | 0 | 0 |
| LYL1 | 0 | 1 | 0 | 0 | 1 | 0 | 0 |
| MAF | 0 | 0 | 0 | 0 | 1 | 0 | 0 |
| MAFB | 0 | 0 | 0 | 0 | 1 | 1 | 0 |
| MAFF | 0 | 0 | 1 | 1 | 1 | 1 | 0 |
| MAFG | 0 | 1 | 0 | 0 | 1 | 0 | 0 |
| MAFK | 0 | 1 | 1 | 0 | 1 | 1 | 0 |
| MAPK14 | 0 | 0 | 0 | 0 | 1 | 0 | 0 |
| MAU2 | 0 | 1 | 0 | 0 | 0 | 0 | 0 |
| MAX | 0 | 1 | 1 | 0 | 1 | 1 | 0 |
| MAZ | 0 | 1 | 1 | 0 | 1 | 1 | 0 |
| MBD1 | 0 | 1 | 0 | 0 | 1 | 0 | 0 |
| MBD2 | 0 | 0 | 0 | 0 | 1 | 0 | 0 |
| MBD3 | 0 | 1 | 0 | 0 | 0 | 0 | 0 |
| MBD4 | 0 | 1 | 1 | 0 | 1 | 0 | 0 |
| MBD5 | 0 | 1 | 0 | 0 | 0 | 0 | 0 |
| MBL2 | 0 | 0 | 0 | 0 | 1 | 0 | 0 |
| MBTD1 | 0 | 0 | 0 | 0 | 1 | 0 | 0 |
| MBTPS2 | 0 | 0 | 0 | 0 | 1 | 0 | 0 |
| MCM2 | 0 | 1 | 0 | 0 | 0 | 0 | 0 |
| MCM7 | 0 | 0 | 0 | 0 | 1 | 0 | 0 |
| ME1 | 0 | 0 | 0 | 0 | 1 | 0 | 0 |
| ME3 | 0 | 0 | 0 | 0 | 1 | 0 | 0 |
| MEAF6 | 0 | 1 | 0 | 0 | 0 | 0 | 0 |
| MECOM | 0 | 1 | 0 | 0 | 1 | 1 | 0 |
| MECP2 | 0 | 0 | 0 | 0 | 1 | 0 | 0 |
| MED1 | 0 | 1 | 0 | 0 | 0 | 1 | 0 |
| MED12 | 0 | 1 | 0 | 0 | 1 | 1 | 0 |
| MED13 | 0 | 1 | 0 | 0 | 0 | 0 | 0 |
| MED26 | 0 | 1 | 0 | 0 | 1 | 0 | 0 |
| MEF2A | 0 | 1 | 1 | 1 | 1 | 1 | 0 |
| MEF2B | 0 | 1 | 0 | 1 | 1 | 0 | 0 |
| MEF2C | 0 | 1 | 1 | 1 | 1 | 0 | 0 |
| MEF2D | 0 | 1 | 0 | 1 | 0 | 0 | 0 |
| MEIS1 | 0 | 1 | 0 | 0 | 1 | 0 | 0 |
| MEIS2 | 0 | 1 | 0 | 1 | 1 | 0 | 0 |
| MEIS3P1 | 0 | 0 | 0 | 0 | 1 | 0 | 0 |
| MEN1 | 0 | 1 | 0 | 0 | 1 | 0 | 0 |
| MESP1 | 0 | 1 | 0 | 0 | 0 | 0 | 0 |
| MGA | 0 | 1 | 0 | 0 | 0 | 0 | 0 |
| MGA::EVX1 | 0 | 0 | 0 | 1 | 0 | 0 | 0 |
| MGMT | 0 | 1 | 0 | 0 | 0 | 0 | 0 |
| MIER2 | 0 | 1 | 0 | 0 | 1 | 0 | 0 |
| MIER3 | 0 | 1 | 0 | 0 | 1 | 0 | 0 |
| MIF | 0 | 1 | 0 | 0 | 0 | 0 | 0 |
| MITF | 1 | 1 | 0 | 0 | 1 | 0 | 0 |
| MIXL1 | 0 | 1 | 0 | 0 | 1 | 0 | 0 |
| MLLT1 | 0 | 1 | 0 | 0 | 1 | 0 | 0 |
| MLLT3 | 0 | 0 | 0 | 0 | 1 | 0 | 0 |
| MLLT6 | 0 | 1 | 0 | 0 | 0 | 0 | 0 |
| MLX | 0 | 0 | 0 | 0 | 1 | 0 | 0 |
| MLXIP | 0 | 0 | 0 | 0 | 1 | 0 | 0 |
| MNT | 0 | 1 | 0 | 0 | 1 | 1 | 0 |
| MNX1 | 0 | 1 | 0 | 0 | 0 | 0 | 0 |
| MORC2 | 0 | 0 | 0 | 0 | 1 | 0 | 0 |
| MRE11A | 0 | 0 | 0 | 0 | 0 | 1 | 0 |
| MSC | 0 | 0 | 0 | 0 | 1 | 0 | 0 |
| MTA1 | 0 | 1 | 0 | 0 | 1 | 0 | 0 |
| MTA2 | 0 | 1 | 0 | 0 | 1 | 0 | 0 |
| MTA3 | 0 | 1 | 1 | 0 | 1 | 0 | 0 |
| MTOR | 0 | 1 | 0 | 0 | 0 | 0 | 0 |
| MUC22 | 0 | 0 | 0 | 0 | 1 | 0 | 0 |
| MXD1 | 0 | 1 | 0 | 0 | 0 | 0 | 0 |
| MXD3 | 0 | 1 | 0 | 0 | 1 | 0 | 0 |
| MXD4 | 0 | 1 | 0 | 0 | 1 | 0 | 0 |
| MXI1 | 0 | 1 | 1 | 0 | 1 | 1 | 0 |
| MYB | 0 | 1 | 1 | 0 | 1 | 1 | 0 |
| MYBL2 | 0 | 1 | 1 | 0 | 1 | 1 | 1 |
| MYC | 1 | 1 | 1 | 0 | 1 | 1 | 0 |
| MYCN | 1 | 1 | 0 | 0 | 1 | 0 | 0 |
| MYF5 | 0 | 1 | 0 | 0 | 0 | 0 | 0 |
| MYH11 | 0 | 0 | 0 | 0 | 1 | 1 | 0 |
| MYNN | 0 | 0 | 0 | 0 | 1 | 0 | 0 |
| MYOCD | 0 | 1 | 0 | 0 | 0 | 0 | 0 |
| MYOD1 | 0 | 1 | 0 | 0 | 1 | 0 | 0 |
| MYOG | 0 | 0 | 1 | 0 | 1 | 0 | 0 |
| MYRF | 0 | 0 | 0 | 0 | 1 | 0 | 0 |
| NAB2 | 0 | 0 | 0 | 0 | 1 | 0 | 0 |
| NABP2 | 0 | 1 | 0 | 0 | 0 | 0 | 0 |
| NAIF1 | 0 | 1 | 0 | 0 | 0 | 0 | 0 |
| NANOG | 1 | 1 | 1 | 0 | 1 | 1 | 0 |
| NBN | 0 | 1 | 0 | 0 | 1 | 0 | 0 |
| NCAPH2 | 0 | 0 | 0 | 0 | 1 | 0 | 0 |
| NCBP1 | 0 | 1 | 0 | 0 | 0 | 0 | 0 |
| NCOA1 | 0 | 1 | 0 | 0 | 1 | 0 | 0 |
| NCOA3 | 0 | 1 | 0 | 0 | 1 | 0 | 0 |
| NCOA6 | 0 | 1 | 0 | 0 | 0 | 0 | 0 |
| NCOR1 | 0 | 1 | 1 | 0 | 1 | 1 | 0 |
| NCOR2 | 0 | 0 | 0 | 0 | 1 | 0 | 0 |
| NELFA | 0 | 1 | 0 | 0 | 1 | 0 | 0 |
| NELFCD | 0 | 1 | 0 | 0 | 0 | 0 | 0 |
| NELFE | 0 | 1 | 1 | 0 | 1 | 0 | 0 |
| NETO2 | 0 | 1 | 0 | 0 | 0 | 0 | 0 |
| NEUROD1 | 0 | 1 | 0 | 1 | 1 | 0 | 0 |
| NEUROG2 | 0 | 0 | 0 | 1 | 1 | 0 | 0 |
| NFAT5 | 0 | 1 | 0 | 0 | 0 | 0 | 0 |
| NFATC1 | 0 | 0 | 1 | 0 | 1 | 0 | 0 |
| NFATC2 | 0 | 0 | 0 | 0 | 1 | 0 | 0 |
| NFATC3 | 0 | 0 | 0 | 0 | 1 | 0 | 0 |
| NFE2 | 0 | 1 | 1 | 0 | 1 | 1 | 0 |
| NFE2L1 | 0 | 0 | 0 | 0 | 1 | 0 | 0 |
| NFE2L2 | 0 | 1 | 0 | 0 | 1 | 1 | 0 |
| NFIA | 0 | 1 | 0 | 0 | 1 | 0 | 1 |
| NFIB | 0 | 1 | 0 | 0 | 0 | 0 | 0 |
| NFIC | 0 | 1 | 1 | 1 | 1 | 1 | 0 |
| NFIL3 | 0 | 1 | 0 | 0 | 1 | 0 | 0 |
| NFIX | 0 | 0 | 0 | 0 | 0 | 0 | 1 |
| NFKB1 | 0 | 1 | 0 | 0 | 1 | 0 | 0 |
| NFKB2 | 0 | 1 | 0 | 0 | 1 | 0 | 0 |
| NFKBIZ | 0 | 1 | 0 | 0 | 1 | 0 | 0 |
| NFRKB | 0 | 0 | 0 | 0 | 1 | 0 | 0 |
| NFYA | 0 | 0 | 0 | 1 | 1 | 1 | 0 |
| NFYB | 0 | 0 | 0 | 0 | 1 | 1 | 0 |
| NFYC | 0 | 0 | 0 | 0 | 1 | 0 | 0 |
| NIPBL | 0 | 1 | 0 | 0 | 1 | 1 | 0 |
| NKRF | 0 | 1 | 0 | 0 | 0 | 0 | 0 |
| NKX2-1 | 0 | 1 | 0 | 0 | 1 | 1 | 0 |
| NKX2-2 | 0 | 1 | 0 | 0 | 0 | 0 | 0 |
| NKX2-8 | 0 | 0 | 0 | 1 | 0 | 0 | 0 |
| NKX3-1 | 0 | 1 | 0 | 0 | 0 | 1 | 0 |
| NONO | 0 | 1 | 0 | 0 | 1 | 0 | 0 |
| NOTCH1 | 0 | 1 | 0 | 0 | 1 | 1 | 0 |
| NPM1 | 0 | 1 | 0 | 0 | 0 | 0 | 0 |
| NR0B1 | 1 | 0 | 0 | 0 | 1 | 0 | 0 |
| NR0B2 | 0 | 1 | 0 | 0 | 0 | 0 | 0 |
| NR1H2 | 0 | 1 | 0 | 0 | 1 | 0 | 0 |
| NR1H3 | 0 | 1 | 0 | 0 | 1 | 0 | 0 |
| NR2C1 | 0 | 0 | 0 | 0 | 1 | 0 | 0 |
| NR2C2 | 0 | 1 | 0 | 1 | 1 | 1 | 1 |
| NR2F1 | 0 | 1 | 0 | 1 | 1 | 0 | 1 |
| NR2F2 | 0 | 1 | 1 | 1 | 1 | 1 | 1 |
| NR2F6 | 0 | 1 | 0 | 0 | 1 | 0 | 0 |
| NR3C1 | 0 | 1 | 1 | 0 | 1 | 1 | 1 |
| NR3C2 | 0 | 0 | 0 | 0 | 0 | 0 | 1 |
| NR4A1 | 0 | 0 | 0 | 1 | 1 | 0 | 1 |
| NR4A2 | 0 | 0 | 0 | 1 | 0 | 0 | 1 |
| NR5A1 | 0 | 1 | 0 | 1 | 0 | 0 | 0 |
| NR5A2 | 0 | 0 | 0 | 0 | 1 | 0 | 0 |
| NR6A1 | 0 | 0 | 0 | 1 | 0 | 0 | 0 |
| NRF1 | 0 | 1 | 1 | 0 | 1 | 1 | 0 |
| NRIP1 | 0 | 1 | 0 | 0 | 0 | 1 | 0 |
| NRL | 0 | 1 | 0 | 1 | 0 | 0 | 1 |
| NSD2 | 0 | 0 | 0 | 0 | 1 | 0 | 0 |
| NUP153 | 0 | 0 | 0 | 0 | 1 | 0 | 0 |
| NUP98 | 0 | 0 | 0 | 0 | 1 | 0 | 0 |
| NUTM1 | 0 | 1 | 0 | 0 | 0 | 0 | 0 |
| OGG1 | 0 | 0 | 0 | 0 | 1 | 0 | 0 |
| OGT | 0 | 1 | 0 | 0 | 0 | 0 | 0 |
| OLIG2 | 0 | 1 | 0 | 0 | 1 | 0 | 0 |
| ONECUT2 | 0 | 0 | 0 | 0 | 1 | 0 | 0 |
| OR2M7 | 0 | 0 | 0 | 0 | 1 | 0 | 0 |
| ORC2 | 0 | 1 | 0 | 0 | 1 | 0 | 0 |
| OSR1 | 0 | 0 | 0 | 1 | 0 | 0 | 0 |
| OSR2 | 0 | 1 | 0 | 1 | 1 | 0 | 0 |
| OTX2 | 0 | 1 | 0 | 0 | 1 | 1 | 0 |
| OVOL1 | 0 | 1 | 0 | 1 | 0 | 0 | 1 |
| OVOL2 | 0 | 0 | 0 | 1 | 1 | 0 | 1 |
| p65 | 0 | 0 | 0 | 0 | 1 | 0 | 0 |
| PADI2 | 0 | 0 | 0 | 0 | 1 | 0 | 0 |
| PAF1 | 0 | 1 | 0 | 0 | 0 | 0 | 0 |
| PALB2 | 0 | 0 | 0 | 0 | 1 | 0 | 0 |
| PARK7 | 0 | 1 | 0 | 0 | 0 | 0 | 0 |
| PARP1 | 0 | 0 | 0 | 0 | 1 | 0 | 0 |
| PATZ1 | 0 | 1 | 0 | 1 | 1 | 0 | 0 |
| PAX1 | 0 | 0 | 0 | 1 | 0 | 0 | 1 |
| PAX2 | 0 | 0 | 0 | 1 | 0 | 0 | 1 |
| PAX5 | 0 | 1 | 1 | 1 | 1 | 1 | 0 |
| PAX8 | 0 | 0 | 0 | 1 | 0 | 0 | 0 |
| PAX9 | 0 | 0 | 0 | 0 | 0 | 0 | 1 |
| PBRM1 | 0 | 1 | 0 | 0 | 0 | 0 | 0 |
| PBX1 | 0 | 1 | 0 | 0 | 1 | 1 | 0 |
| PBX2 | 0 | 0 | 0 | 0 | 1 | 0 | 0 |
| PBX3 | 0 | 1 | 1 | 1 | 1 | 1 | 1 |
| PBX4 | 0 | 1 | 0 | 0 | 1 | 0 | 0 |
| PBXIP1 | 0 | 1 | 0 | 0 | 0 | 0 | 0 |
| PCBP1 | 0 | 1 | 0 | 0 | 1 | 0 | 0 |
| PCBP2 | 0 | 0 | 0 | 0 | 1 | 0 | 0 |
| PCF11 | 0 | 0 | 0 | 0 | 1 | 0 | 0 |
| PDS5A | 0 | 1 | 0 | 0 | 0 | 0 | 0 |
| PDS5B | 0 | 1 | 0 | 0 | 0 | 0 | 0 |
| PDX1 | 0 | 1 | 0 | 0 | 1 | 0 | 0 |
| PEX2 | 0 | 0 | 0 | 0 | 1 | 0 | 0 |
| PGR | 0 | 1 | 0 | 1 | 1 | 1 | 1 |
| PHF2 | 0 | 1 | 0 | 0 | 0 | 0 | 0 |
| PHF20 | 0 | 1 | 0 | 0 | 0 | 0 | 0 |
| PHF21A | 0 | 1 | 0 | 0 | 0 | 0 | 0 |
| PHF5A | 0 | 1 | 0 | 0 | 1 | 0 | 0 |
| PHF6 | 0 | 0 | 0 | 0 | 1 | 0 | 0 |
| PHF8 | 0 | 1 | 1 | 0 | 1 | 1 | 0 |
| PHIP | 0 | 1 | 0 | 0 | 0 | 0 | 0 |
| PHOX2B | 0 | 0 | 0 | 1 | 1 | 0 | 0 |
| PIAS1 | 0 | 1 | 0 | 0 | 1 | 0 | 0 |
| PITX1 | 0 | 1 | 0 | 0 | 0 | 0 | 0 |
| PKNOX1 | 0 | 1 | 0 | 1 | 1 | 0 | 0 |
| PLAG1 | 0 | 1 | 0 | 0 | 0 | 0 | 0 |
| PLSCR1 | 0 | 1 | 0 | 0 | 0 | 0 | 0 |
| PMEPA1 | 0 | 0 | 0 | 0 | 1 | 0 | 0 |
| PML | 0 | 1 | 1 | 0 | 1 | 1 | 0 |
| POGZ | 0 | 1 | 0 | 0 | 0 | 0 | 0 |
| POLR2A | 0 | 0 | 1 | 0 | 0 | 1 | 0 |
| POLR2C | 0 | 1 | 0 | 0 | 0 | 0 | 0 |
| POLR3A | 0 | 0 | 0 | 0 | 0 | 1 | 0 |
| POU2AF1 | 0 | 1 | 0 | 0 | 0 | 0 | 0 |
| POU2F1 | 0 | 1 | 0 | 0 | 1 | 1 | 0 |
| POU2F2 | 0 | 1 | 1 | 0 | 1 | 0 | 0 |
| POU2F3 | 0 | 1 | 0 | 1 | 0 | 0 | 0 |
| POU3F2 | 0 | 0 | 0 | 0 | 1 | 0 | 0 |
| POU4F1 | 0 | 0 | 0 | 1 | 0 | 0 | 0 |
| POU4F3 | 0 | 0 | 0 | 1 | 0 | 0 | 0 |
| POU5F1 | 1 | 1 | 0 | 1 | 1 | 1 | 0 |
| PPARA | 0 | 0 | 0 | 0 | 1 | 0 | 0 |
| PPARA::RXRA | 0 | 0 | 0 | 1 | 0 | 0 | 1 |
| PPARD | 0 | 0 | 0 | 1 | 1 | 0 | 1 |
| PPARG | 1 | 1 | 0 | 0 | 1 | 1 | 0 |
| PPARGC1A | 0 | 0 | 0 | 0 | 0 | 1 | 0 |
| PRAME | 0 | 1 | 0 | 0 | 0 | 0 | 0 |
| PRDM1 | 0 | 1 | 1 | 0 | 1 | 1 | 0 |
| PRDM10 | 0 | 1 | 0 | 0 | 1 | 0 | 0 |
| PRDM11 | 0 | 0 | 0 | 0 | 1 | 0 | 0 |
| PRDM14 | 1 | 0 | 0 | 0 | 1 | 0 | 0 |
| PRDM4 | 0 | 0 | 0 | 0 | 1 | 0 | 0 |
| PRDM6 | 0 | 1 | 0 | 0 | 1 | 0 | 0 |
| PRDM9 | 0 | 0 | 0 | 1 | 1 | 0 | 0 |
| PRMT1 | 0 | 1 | 0 | 0 | 1 | 0 | 0 |
| PRMT5 | 0 | 0 | 0 | 0 | 1 | 0 | 0 |
| PROSER1 | 0 | 1 | 0 | 0 | 0 | 0 | 0 |
| PROX1 | 0 | 1 | 0 | 0 | 0 | 0 | 0 |
| PRPF4 | 0 | 1 | 0 | 0 | 1 | 0 | 0 |
| PTBP1 | 0 | 1 | 0 | 0 | 1 | 0 | 0 |
| PTEN | 0 | 0 | 0 | 0 | 1 | 0 | 0 |
| PYGO2 | 0 | 0 | 0 | 0 | 1 | 0 | 0 |
| QRICH1 | 0 | 1 | 0 | 0 | 0 | 0 | 0 |
| QSER1 | 0 | 1 | 0 | 0 | 0 | 0 | 0 |
| RAC1 | 0 | 1 | 0 | 0 | 0 | 0 | 0 |
| RAD21 | 0 | 1 | 1 | 0 | 1 | 1 | 0 |
| RAG1 | 0 | 0 | 0 | 0 | 1 | 0 | 0 |
| RAG2 | 0 | 1 | 0 | 0 | 1 | 0 | 0 |
| RARA | 0 | 1 | 0 | 0 | 1 | 1 | 0 |
| RARA::RXRG | 0 | 0 | 0 | 1 | 0 | 0 | 0 |
| RARG | 0 | 0 | 0 | 0 | 0 | 1 | 0 |
| RB1 | 0 | 0 | 0 | 0 | 1 | 1 | 0 |
| RBAK | 0 | 1 | 0 | 0 | 1 | 0 | 0 |
| RBBP4 | 0 | 0 | 0 | 0 | 1 | 0 | 0 |
| RBBP5 | 0 | 1 | 1 | 0 | 1 | 1 | 0 |
| RBBP7 | 0 | 1 | 0 | 0 | 0 | 0 | 0 |
| RBCK1 | 0 | 0 | 0 | 0 | 0 | 1 | 0 |
| RBFOX2 | 0 | 1 | 0 | 0 | 1 | 0 | 0 |
| RBL1 | 0 | 1 | 0 | 0 | 0 | 0 | 0 |
| RBL2 | 0 | 0 | 0 | 0 | 1 | 1 | 0 |
| RBM14 | 0 | 0 | 0 | 0 | 1 | 0 | 0 |
| RBM22 | 0 | 1 | 0 | 0 | 1 | 0 | 0 |
| RBM25 | 0 | 1 | 0 | 0 | 1 | 0 | 0 |
| RBM34 | 0 | 0 | 0 | 0 | 1 | 0 | 0 |
| RBM39 | 0 | 1 | 0 | 0 | 1 | 0 | 0 |
| RBPJ | 0 | 1 | 0 | 0 | 1 | 1 | 1 |
| RCOR1 | 0 | 1 | 1 | 0 | 1 | 1 | 0 |
| RCOR2 | 0 | 0 | 0 | 0 | 1 | 0 | 0 |
| REL | 0 | 1 | 0 | 0 | 1 | 0 | 0 |
| RELA | 1 | 1 | 1 | 0 | 1 | 1 | 0 |
| RELB | 0 | 1 | 0 | 0 | 1 | 0 | 0 |
| RERE | 0 | 1 | 0 | 0 | 1 | 0 | 0 |
| REST | 0 | 1 | 1 | 0 | 1 | 1 | 0 |
| RFX1 | 0 | 0 | 0 | 0 | 1 | 0 | 0 |
| RFX5 | 0 | 0 | 1 | 0 | 1 | 0 | 0 |
| RFXANK | 0 | 1 | 0 | 0 | 1 | 0 | 0 |
| RFXAP | 0 | 1 | 0 | 0 | 0 | 0 | 0 |
| RING1 | 0 | 0 | 0 | 0 | 1 | 0 | 0 |
| RNF2 | 0 | 1 | 1 | 0 | 1 | 1 | 0 |
| RNGTT | 0 | 0 | 0 | 0 | 1 | 0 | 0 |
| RORA | 0 | 1 | 0 | 1 | 0 | 0 | 1 |
| RORC | 0 | 1 | 0 | 1 | 1 | 0 | 0 |
| RREB1 | 0 | 1 | 0 | 0 | 0 | 0 | 0 |
| RUNX1 | 1 | 1 | 0 | 0 | 1 | 1 | 0 |
| RUNX1T1 | 0 | 1 | 0 | 0 | 1 | 1 | 0 |
| RUNX2 | 0 | 1 | 0 | 1 | 1 | 1 | 1 |
| RUNX3 | 0 | 1 | 1 | 0 | 1 | 1 | 0 |
| RUVBL2 | 0 | 1 | 0 | 0 | 0 | 0 | 0 |
| RXRA | 0 | 1 | 1 | 0 | 1 | 1 | 0 |
| RXRB | 0 | 1 | 0 | 1 | 1 | 0 | 1 |
| RXRG | 0 | 0 | 0 | 1 | 0 | 1 | 1 |
| RYBP | 0 | 0 | 0 | 0 | 0 | 1 | 0 |
| SAFB | 0 | 1 | 0 | 0 | 1 | 0 | 0 |
| SALL3 | 0 | 0 | 0 | 0 | 1 | 0 | 0 |
| SALL4 | 1 | 0 | 0 | 0 | 0 | 0 | 0 |
| SAP130 | 0 | 1 | 0 | 0 | 1 | 0 | 0 |
| SAP30 | 0 | 1 | 1 | 0 | 1 | 1 | 0 |
| SATB1 | 0 | 0 | 0 | 0 | 1 | 0 | 0 |
| SATB2 | 0 | 1 | 0 | 0 | 0 | 0 | 0 |
| SCML2 | 0 | 0 | 0 | 0 | 1 | 0 | 0 |
| SCRT1 | 0 | 0 | 0 | 0 | 1 | 0 | 0 |
| SCRT2 | 0 | 1 | 0 | 0 | 1 | 0 | 0 |
| SETD1A | 0 | 1 | 0 | 0 | 0 | 0 | 0 |
| SETDB1 | 0 | 0 | 0 | 0 | 1 | 1 | 0 |
| SFMBT1 | 0 | 0 | 0 | 0 | 0 | 1 | 0 |
| SFPQ | 0 | 1 | 0 | 0 | 1 | 0 | 0 |
| SIN3A | 0 | 1 | 1 | 0 | 1 | 1 | 0 |
| SIN3B | 0 | 1 | 0 | 0 | 1 | 0 | 0 |
| SIRT6 | 0 | 0 | 1 | 0 | 1 | 0 | 0 |
| SIX1 | 0 | 0 | 0 | 1 | 0 | 0 | 0 |
| SIX2 | 0 | 1 | 0 | 1 | 1 | 0 | 0 |
| SIX4 | 0 | 1 | 0 | 0 | 0 | 0 | 0 |
| SIX5 | 0 | 0 | 1 | 0 | 1 | 1 | 0 |
| SKI | 0 | 1 | 0 | 0 | 1 | 0 | 0 |
| SKIL | 0 | 1 | 0 | 0 | 0 | 0 | 0 |
| SLC30A9 | 0 | 0 | 0 | 0 | 1 | 0 | 0 |
| SMAD1 | 0 | 1 | 0 | 0 | 1 | 1 | 0 |
| SMAD2 | 0 | 1 | 0 | 0 | 1 | 0 | 0 |
| SMAD2/SMAD3 | 0 | 0 | 0 | 0 | 0 | 1 | 0 |
| SMAD3 | 0 | 1 | 0 | 0 | 1 | 0 | 0 |
| SMAD4 | 1 | 1 | 0 | 0 | 1 | 1 | 0 |
| SMAD5 | 0 | 1 | 0 | 0 | 1 | 0 | 0 |
| SMARCA2 | 0 | 1 | 0 | 0 | 1 | 0 | 0 |
| SMARCA4 | 1 | 1 | 0 | 0 | 1 | 1 | 0 |
| SMARCA5 | 0 | 1 | 0 | 0 | 1 | 0 | 0 |
| SMARCB1 | 0 | 1 | 1 | 0 | 1 | 0 | 0 |
| SMARCC1 | 0 | 1 | 0 | 0 | 1 | 0 | 0 |
| SMARCC2 | 0 | 1 | 0 | 0 | 1 | 0 | 0 |
| SMARCE1 | 0 | 1 | 0 | 0 | 1 | 0 | 0 |
| SMC1A | 0 | 1 | 0 | 0 | 1 | 1 | 0 |
| SMC3 | 0 | 1 | 1 | 0 | 1 | 1 | 0 |
| SNAI1 | 0 | 0 | 0 | 0 | 1 | 0 | 0 |
| SNAI2 | 0 | 1 | 0 | 0 | 1 | 1 | 0 |
| SNRNP70 | 0 | 0 | 0 | 0 | 1 | 0 | 0 |
| SON | 0 | 0 | 0 | 0 | 1 | 0 | 0 |
| SOX10 | 0 | 1 | 0 | 0 | 0 | 0 | 0 |
| SOX11 | 1 | 0 | 0 | 0 | 0 | 0 | 0 |
| SOX13 | 0 | 1 | 0 | 0 | 1 | 0 | 0 |
| SOX17 | 0 | 0 | 0 | 0 | 1 | 0 | 0 |
| SOX2 | 1 | 1 | 0 | 0 | 1 | 1 | 0 |
| SOX4 | 0 | 0 | 0 | 1 | 1 | 0 | 0 |
| SOX5 | 0 | 1 | 0 | 0 | 1 | 0 | 0 |
| SOX6 | 0 | 1 | 0 | 0 | 0 | 0 | 0 |
| SOX8 | 0 | 0 | 0 | 0 | 1 | 0 | 0 |
| SOX9 | 0 | 0 | 0 | 0 | 1 | 0 | 0 |
| SP1 | 0 | 1 | 1 | 1 | 1 | 1 | 0 |
| SP140 | 0 | 0 | 0 | 0 | 1 | 0 | 0 |
| SP140L | 0 | 1 | 0 | 0 | 0 | 0 | 0 |
| SP2 | 0 | 1 | 0 | 1 | 1 | 0 | 0 |
| SP3 | 0 | 1 | 0 | 1 | 1 | 0 | 0 |
| SP4 | 0 | 1 | 1 | 0 | 1 | 1 | 0 |
| SP5 | 0 | 1 | 0 | 0 | 1 | 0 | 0 |
| SP7 | 0 | 1 | 0 | 0 | 0 | 0 | 0 |
| SP8 | 0 | 0 | 0 | 1 | 0 | 0 | 0 |
| SP9 | 0 | 0 | 0 | 1 | 0 | 0 | 0 |
| SPDEF | 0 | 0 | 0 | 0 | 1 | 1 | 0 |
| SPEN | 0 | 1 | 0 | 0 | 0 | 0 | 0 |
| SPI1 | 1 | 1 | 1 | 0 | 1 | 1 | 0 |
| SPIB | 0 | 0 | 0 | 1 | 1 | 0 | 0 |
| SRC | 0 | 0 | 0 | 0 | 1 | 1 | 0 |
| SREBF1 | 0 | 0 | 0 | 0 | 1 | 0 | 0 |
| SREBF2 | 0 | 1 | 0 | 0 | 1 | 0 | 0 |
| SRF | 1 | 1 | 1 | 0 | 1 | 1 | 0 |
| SRSF1 | 0 | 1 | 0 | 0 | 1 | 0 | 0 |
| SRSF3 | 0 | 1 | 0 | 0 | 1 | 0 | 0 |
| SRSF4 | 0 | 1 | 0 | 0 | 1 | 0 | 0 |
| SRSF7 | 0 | 0 | 0 | 0 | 1 | 0 | 0 |
| SRSF9 | 0 | 0 | 0 | 0 | 1 | 0 | 0 |
| SS18 | 0 | 1 | 0 | 0 | 1 | 0 | 0 |
| SS18L1 | 0 | 1 | 0 | 0 | 0 | 0 | 0 |
| SSRP1 | 0 | 1 | 0 | 0 | 1 | 0 | 0 |
| SSU72 | 0 | 0 | 0 | 0 | 1 | 0 | 0 |
| STAG1 | 0 | 1 | 0 | 0 | 1 | 1 | 0 |
| STAG2 | 0 | 1 | 0 | 0 | 1 | 0 | 0 |
| STAT1 | 0 | 1 | 1 | 1 | 1 | 1 | 0 |
| STAT2 | 0 | 1 | 0 | 0 | 1 | 0 | 0 |
| STAT3 | 0 | 1 | 1 | 1 | 1 | 1 | 0 |
| STAT4 | 0 | 0 | 0 | 0 | 1 | 0 | 0 |
| STAT5A | 0 | 1 | 1 | 0 | 1 | 1 | 0 |
| STAT5B | 0 | 0 | 0 | 0 | 1 | 1 | 0 |
| STAT6 | 0 | 0 | 0 | 0 | 1 | 0 | 0 |
| SUMO1 | 0 | 1 | 0 | 0 | 0 | 0 | 0 |
| SUMO2 | 0 | 1 | 0 | 0 | 1 | 1 | 0 |
| SUPT16H | 0 | 0 | 0 | 0 | 1 | 0 | 0 |
| SUPT5H | 0 | 1 | 0 | 0 | 1 | 0 | 0 |
| SUPT6H | 0 | 1 | 0 | 0 | 1 | 0 | 0 |
| SUZ12 | 0 | 1 | 1 | 0 | 1 | 1 | 0 |
| T-Cell | 0 | 0 | 0 | 0 | 1 | 0 | 0 |
| TAF1 | 0 | 1 | 1 | 0 | 1 | 1 | 0 |
| TAF15 | 0 | 0 | 0 | 0 | 1 | 0 | 0 |
| TAF3 | 0 | 0 | 0 | 0 | 1 | 1 | 0 |
| TAF7 | 0 | 0 | 1 | 0 | 1 | 1 | 0 |
| TAF9B | 0 | 1 | 0 | 0 | 0 | 0 | 0 |
| TAL1 | 1 | 1 | 1 | 0 | 1 | 1 | 0 |
| TAL1::TCF3 | 0 | 0 | 0 | 1 | 0 | 0 | 0 |
| TARDBP | 0 | 1 | 0 | 0 | 1 | 0 | 0 |
| TBL1X | 0 | 0 | 0 | 0 | 0 | 1 | 0 |
| TBL1XR1 | 0 | 1 | 1 | 0 | 1 | 1 | 0 |
| TBP | 0 | 1 | 1 | 0 | 1 | 0 | 0 |
| TBPL1 | 0 | 1 | 0 | 0 | 0 | 0 | 0 |
| TBX15 | 0 | 0 | 0 | 1 | 0 | 0 | 0 |
| TBX18 | 0 | 0 | 0 | 1 | 0 | 0 | 0 |
| TBX2 | 0 | 0 | 0 | 0 | 1 | 0 | 0 |
| TBX20 | 0 | 0 | 0 | 1 | 0 | 0 | 0 |
| TBX21 | 0 | 1 | 0 | 1 | 1 | 1 | 0 |
| TBX3 | 0 | 1 | 0 | 0 | 0 | 0 | 0 |
| TBX5 | 0 | 0 | 0 | 1 | 1 | 0 | 0 |
| TCF12 | 0 | 1 | 1 | 0 | 1 | 1 | 0 |
| TCF21 | 0 | 1 | 0 | 0 | 1 | 1 | 0 |
| TCF25 | 0 | 1 | 0 | 0 | 0 | 0 | 0 |
| TCF3 | 1 | 1 | 1 | 0 | 1 | 1 | 0 |
| TCF4 | 0 | 0 | 0 | 1 | 1 | 0 | 0 |
| TCF7 | 0 | 1 | 0 | 1 | 1 | 0 | 0 |
| TCF7L1 | 0 | 0 | 0 | 0 | 1 | 0 | 0 |
| TCF7L2 | 0 | 1 | 1 | 0 | 1 | 1 | 0 |
| TCFL5 | 0 | 0 | 0 | 1 | 0 | 0 | 0 |
| TEAD1 | 0 | 1 | 0 | 0 | 1 | 0 | 0 |
| TEAD3 | 0 | 1 | 0 | 0 | 1 | 0 | 0 |
| TEAD4 | 0 | 1 | 1 | 0 | 1 | 1 | 0 |
| TEF | 0 | 1 | 0 | 0 | 0 | 0 | 0 |
| TET1 | 0 | 0 | 0 | 0 | 1 | 0 | 0 |
| TET2 | 0 | 1 | 0 | 0 | 1 | 0 | 0 |
| TFAP2A | 0 | 1 | 0 | 0 | 1 | 1 | 0 |
| TFAP2B | 0 | 1 | 0 | 1 | 0 | 0 | 0 |
| TFAP2C | 1 | 1 | 0 | 1 | 1 | 1 | 0 |
| TFAP4 | 0 | 1 | 0 | 1 | 1 | 1 | 0 |
| TFAP4::ETV1 | 0 | 0 | 0 | 1 | 0 | 0 | 0 |
| TFCP2L1 | 0 | 1 | 0 | 0 | 0 | 0 | 0 |
| TFDP1 | 0 | 1 | 0 | 1 | 1 | 1 | 0 |
| TFDP2 | 0 | 1 | 0 | 0 | 0 | 0 | 0 |
| TFE3 | 0 | 1 | 0 | 0 | 1 | 0 | 0 |
| TFEB | 0 | 1 | 0 | 0 | 0 | 0 | 0 |
| TGIF2 | 0 | 1 | 0 | 0 | 1 | 0 | 0 |
| TGIF2LX | 0 | 1 | 0 | 0 | 0 | 0 | 0 |
| THAP1 | 0 | 0 | 1 | 1 | 1 | 1 | 0 |
| THAP11 | 0 | 0 | 0 | 0 | 1 | 0 | 0 |
| THAP12 | 0 | 1 | 0 | 0 | 0 | 0 | 0 |
| THAP7 | 0 | 1 | 0 | 0 | 0 | 0 | 0 |
| THAP8 | 0 | 1 | 0 | 0 | 0 | 0 | 0 |
| THAP9 | 0 | 1 | 0 | 0 | 0 | 0 | 0 |
| THRA | 0 | 1 | 0 | 0 | 0 | 0 | 0 |
| THRB | 0 | 1 | 0 | 1 | 1 | 0 | 1 |
| TIGD6 | 0 | 1 | 0 | 0 | 0 | 0 | 0 |
| TLE3 | 0 | 1 | 0 | 0 | 1 | 0 | 0 |
| TLX1 | 0 | 1 | 0 | 0 | 1 | 0 | 0 |
| TLX3 | 0 | 1 | 0 | 0 | 0 | 0 | 0 |
| TOE1 | 0 | 1 | 0 | 0 | 0 | 0 | 0 |
| TOP1 | 0 | 0 | 0 | 0 | 1 | 0 | 0 |
| TOP2B | 0 | 1 | 0 | 0 | 1 | 0 | 0 |
| TOX4 | 0 | 1 | 0 | 0 | 0 | 0 | 0 |
| TP53 | 1 | 1 | 0 | 0 | 1 | 1 | 0 |
| TP53BP1 | 0 | 1 | 0 | 0 | 1 | 0 | 0 |
| TP63 | 1 | 1 | 0 | 0 | 1 | 1 | 0 |
| TP73 | 0 | 0 | 0 | 0 | 1 | 1 | 0 |
| TRAFD1 | 0 | 1 | 0 | 0 | 0 | 0 | 0 |
| TRIB3 | 0 | 1 | 0 | 0 | 0 | 0 | 0 |
| TRIM22 | 0 | 1 | 0 | 0 | 1 | 0 | 0 |
| TRIM24 | 0 | 1 | 0 | 0 | 1 | 0 | 0 |
| TRIM25 | 0 | 1 | 0 | 0 | 0 | 0 | 0 |
| TRIM28 | 0 | 1 | 1 | 0 | 1 | 1 | 0 |
| TRPS1 | 0 | 1 | 0 | 0 | 1 | 0 | 0 |
| TSC22D4 | 0 | 0 | 0 | 0 | 1 | 0 | 0 |
| TWIST1 | 0 | 1 | 0 | 0 | 0 | 0 | 0 |
| U2AF1 | 0 | 1 | 0 | 0 | 1 | 0 | 0 |
| U2AF2 | 0 | 0 | 0 | 0 | 1 | 0 | 0 |
| UBN1 | 0 | 0 | 0 | 0 | 1 | 1 | 0 |
| UBP1 | 0 | 1 | 0 | 0 | 1 | 0 | 0 |
| UBTF | 0 | 1 | 1 | 0 | 1 | 1 | 0 |
| UPF1 | 0 | 0 | 0 | 0 | 1 | 0 | 0 |
| USF1 | 0 | 1 | 1 | 0 | 1 | 1 | 0 |
| USF2 | 0 | 1 | 1 | 0 | 1 | 1 | 0 |
| USP7 | 0 | 1 | 0 | 0 | 1 | 0 | 0 |
| VCP | 0 | 1 | 0 | 0 | 0 | 0 | 0 |
| VDR | 0 | 1 | 0 | 0 | 1 | 1 | 0 |
| VEZF1 | 0 | 1 | 0 | 0 | 1 | 1 | 0 |
| VSX2 | 0 | 1 | 0 | 0 | 0 | 0 | 0 |
| WAS | 0 | 1 | 0 | 0 | 0 | 0 | 0 |
| WDR33 | 0 | 1 | 0 | 0 | 0 | 0 | 0 |
| WDR5 | 0 | 1 | 0 | 0 | 1 | 1 | 0 |
| WDR77 | 0 | 1 | 0 | 0 | 0 | 0 | 0 |
| WIZ | 0 | 1 | 0 | 0 | 0 | 0 | 0 |
| WRNIP1 | 0 | 0 | 1 | 0 | 0 | 0 | 0 |
| WT1 | 0 | 0 | 0 | 0 | 1 | 0 | 0 |
| XBP1 | 0 | 0 | 0 | 1 | 1 | 1 | 1 |
| XRCC1 | 0 | 1 | 0 | 0 | 0 | 0 | 0 |
| XRCC4 | 0 | 0 | 0 | 0 | 1 | 0 | 0 |
| XRCC5 | 0 | 1 | 0 | 0 | 1 | 0 | 0 |
| XRCC6 | 0 | 1 | 0 | 0 | 0 | 0 | 0 |
| XRN2 | 0 | 0 | 0 | 0 | 1 | 0 | 0 |
| YAP1 | 0 | 1 | 0 | 0 | 1 | 0 | 0 |
| YEATS4 | 0 | 1 | 0 | 0 | 0 | 0 | 0 |
| YY1 | 0 | 1 | 1 | 0 | 1 | 1 | 0 |
| YY2 | 0 | 0 | 0 | 1 | 1 | 0 | 1 |
| ZBED1 | 0 | 1 | 0 | 0 | 1 | 0 | 0 |
| ZBED4 | 0 | 1 | 0 | 1 | 0 | 0 | 0 |
| ZBED5 | 0 | 1 | 0 | 0 | 1 | 0 | 0 |
| ZBTB10 | 0 | 1 | 0 | 0 | 0 | 0 | 0 |
| ZBTB11 | 0 | 1 | 0 | 1 | 1 | 0 | 0 |
| ZBTB14 | 0 | 1 | 0 | 1 | 1 | 0 | 0 |
| ZBTB16 | 0 | 0 | 0 | 0 | 1 | 0 | 0 |
| ZBTB17 | 0 | 1 | 0 | 0 | 1 | 1 | 0 |
| ZBTB18 | 0 | 0 | 0 | 0 | 1 | 0 | 0 |
| ZBTB2 | 0 | 1 | 0 | 0 | 1 | 0 | 0 |
| ZBTB20 | 0 | 1 | 0 | 0 | 1 | 0 | 0 |
| ZBTB21 | 0 | 1 | 0 | 0 | 0 | 0 | 0 |
| ZBTB24 | 0 | 0 | 0 | 1 | 0 | 0 | 0 |
| ZBTB25 | 0 | 1 | 0 | 0 | 1 | 0 | 0 |
| ZBTB26 | 0 | 1 | 0 | 0 | 1 | 0 | 0 |
| ZBTB32 | 0 | 0 | 0 | 1 | 0 | 0 | 0 |
| ZBTB33 | 0 | 1 | 1 | 0 | 1 | 1 | 0 |
| ZBTB37 | 0 | 1 | 0 | 0 | 0 | 0 | 0 |
| ZBTB38 | 0 | 1 | 0 | 0 | 0 | 0 | 0 |
| ZBTB39 | 0 | 1 | 0 | 0 | 0 | 0 | 0 |
| ZBTB40 | 0 | 1 | 0 | 0 | 1 | 0 | 0 |
| ZBTB42 | 0 | 0 | 0 | 0 | 1 | 0 | 0 |
| ZBTB43 | 0 | 1 | 0 | 0 | 0 | 0 | 0 |
| ZBTB44 | 0 | 1 | 0 | 0 | 0 | 0 | 0 |
| ZBTB48 | 0 | 1 | 0 | 0 | 1 | 0 | 0 |
| ZBTB6 | 0 | 1 | 0 | 0 | 1 | 0 | 0 |
| ZBTB7A | 0 | 1 | 1 | 1 | 1 | 1 | 0 |
| ZBTB7B | 0 | 1 | 0 | 1 | 0 | 0 | 0 |
| ZBTB7C | 0 | 0 | 0 | 1 | 0 | 0 | 0 |
| ZBTB8A | 0 | 0 | 0 | 0 | 1 | 0 | 0 |
| ZBTB9 | 0 | 1 | 0 | 0 | 0 | 0 | 0 |
| ZC3H11A | 0 | 0 | 1 | 0 | 1 | 0 | 0 |
| ZC3H4 | 0 | 1 | 0 | 0 | 0 | 0 | 0 |
| ZC3H8 | 0 | 0 | 0 | 0 | 1 | 0 | 0 |
| ZEB1 | 0 | 1 | 1 | 0 | 1 | 1 | 0 |
| ZEB2 | 0 | 1 | 0 | 0 | 1 | 0 | 0 |
| ZFAT | 0 | 0 | 0 | 0 | 1 | 0 | 0 |
| ZFHX2 | 0 | 1 | 0 | 0 | 1 | 0 | 0 |
| ZFP1 | 0 | 0 | 0 | 0 | 1 | 0 | 0 |
| ZFP2 | 0 | 1 | 0 | 0 | 0 | 0 | 0 |
| ZFP36 | 0 | 0 | 0 | 0 | 1 | 0 | 0 |
| ZFP37 | 0 | 1 | 0 | 0 | 0 | 0 | 0 |
| ZFP42 | 1 | 0 | 0 | 0 | 1 | 1 | 0 |
| ZFP64 | 0 | 0 | 0 | 0 | 1 | 0 | 0 |
| ZFP69B | 0 | 1 | 0 | 0 | 1 | 0 | 0 |
| ZFP82 | 0 | 1 | 0 | 0 | 0 | 0 | 0 |
| ZFP91 | 0 | 1 | 0 | 0 | 0 | 0 | 0 |
| ZFX | 0 | 1 | 0 | 0 | 1 | 0 | 0 |
| ZFY | 0 | 1 | 0 | 0 | 0 | 0 | 0 |
| ZGPAT | 0 | 1 | 0 | 0 | 1 | 0 | 0 |
| ZHX1 | 0 | 0 | 0 | 0 | 1 | 0 | 0 |
| ZHX2 | 0 | 1 | 0 | 0 | 1 | 0 | 0 |
| ZIC2 | 0 | 0 | 0 | 0 | 1 | 0 | 0 |
| ZIC4 | 0 | 0 | 0 | 1 | 0 | 0 | 0 |
| ZIC5 | 0 | 0 | 0 | 0 | 1 | 0 | 0 |
| ZIK1 | 0 | 1 | 0 | 0 | 0 | 0 | 0 |
| ZIM3 | 0 | 0 | 0 | 1 | 0 | 0 | 0 |
| ZKSCAN1 | 0 | 1 | 1 | 1 | 1 | 0 | 1 |
| ZKSCAN3 | 0 | 0 | 0 | 1 | 0 | 0 | 0 |
| ZKSCAN5 | 0 | 0 | 0 | 1 | 0 | 0 | 0 |
| ZKSCAN8 | 0 | 1 | 0 | 0 | 1 | 0 | 0 |
| ZMAT3 | 0 | 1 | 0 | 0 | 0 | 0 | 0 |
| ZMAT5 | 0 | 1 | 0 | 0 | 0 | 0 | 0 |
| ZMIZ1 | 0 | 0 | 1 | 0 | 1 | 1 | 0 |
| ZMYM2 | 0 | 1 | 0 | 0 | 0 | 0 | 0 |
| ZMYM3 | 0 | 1 | 0 | 0 | 1 | 0 | 0 |
| ZMYND8 | 0 | 1 | 0 | 0 | 1 | 0 | 0 |
| ZNF12 | 0 | 1 | 0 | 0 | 0 | 1 | 0 |
| ZNF121 | 0 | 1 | 0 | 0 | 0 | 0 | 0 |
| ZNF124 | 0 | 1 | 0 | 0 | 0 | 0 | 0 |
| ZNF142 | 0 | 1 | 0 | 0 | 0 | 0 | 0 |
| ZNF143 | 0 | 1 | 1 | 0 | 1 | 1 | 0 |
| ZNF146 | 0 | 0 | 0 | 0 | 1 | 0 | 0 |
| ZNF148 | 0 | 1 | 0 | 0 | 1 | 0 | 0 |
| ZNF175 | 0 | 0 | 0 | 0 | 1 | 0 | 0 |
| ZNF18 | 0 | 0 | 0 | 0 | 1 | 0 | 0 |
| ZNF184 | 0 | 0 | 0 | 1 | 0 | 0 | 0 |
| ZNF189 | 0 | 1 | 0 | 1 | 0 | 0 | 0 |
| ZNF2 | 0 | 1 | 0 | 0 | 1 | 0 | 0 |
| ZNF20 | 0 | 1 | 0 | 0 | 0 | 0 | 0 |
| ZNF205 | 0 | 1 | 0 | 0 | 1 | 0 | 0 |
| ZNF213 | 0 | 1 | 0 | 1 | 1 | 0 | 0 |
| ZNF217 | 0 | 0 | 1 | 0 | 0 | 0 | 0 |
| ZNF219 | 0 | 1 | 0 | 0 | 0 | 0 | 0 |
| ZNF221 | 0 | 1 | 0 | 0 | 0 | 0 | 0 |
| ZNF224 | 0 | 1 | 0 | 0 | 0 | 0 | 0 |
| ZNF225 | 0 | 1 | 0 | 0 | 0 | 0 | 0 |
| ZNF230 | 0 | 1 | 0 | 0 | 0 | 0 | 0 |
| ZNF232 | 0 | 1 | 0 | 0 | 0 | 0 | 0 |
| ZNF234 | 0 | 1 | 0 | 0 | 0 | 0 | 0 |
| ZNF24 | 0 | 1 | 0 | 0 | 1 | 0 | 0 |
| ZNF256 | 0 | 1 | 0 | 0 | 0 | 0 | 0 |
| ZNF260 | 0 | 0 | 0 | 0 | 1 | 0 | 0 |
| ZNF263 | 0 | 1 | 1 | 1 | 1 | 1 | 0 |
| ZNF264 | 0 | 1 | 0 | 0 | 0 | 0 | 0 |
| ZNF266 | 0 | 0 | 0 | 0 | 1 | 0 | 0 |
| ZNF274 | 0 | 0 | 1 | 0 | 0 | 0 | 0 |
| ZNF276 | 0 | 1 | 0 | 0 | 0 | 0 | 0 |
| ZNF280B | 0 | 1 | 0 | 0 | 0 | 0 | 0 |
| ZNF280D | 0 | 1 | 0 | 0 | 1 | 0 | 0 |
| ZNF281 | 0 | 1 | 0 | 1 | 1 | 0 | 0 |
| ZNF282 | 0 | 1 | 0 | 0 | 1 | 0 | 0 |
| ZNF296 | 0 | 1 | 0 | 0 | 0 | 0 | 0 |
| ZNF3 | 0 | 0 | 0 | 0 | 1 | 0 | 0 |
| ZNF30 | 0 | 1 | 0 | 0 | 1 | 0 | 0 |
| ZNF316 | 0 | 1 | 0 | 0 | 1 | 0 | 0 |
| ZNF317 | 0 | 1 | 0 | 0 | 0 | 0 | 0 |
| ZNF318 | 0 | 1 | 0 | 0 | 1 | 0 | 0 |
| ZNF324 | 0 | 0 | 0 | 0 | 1 | 0 | 0 |
| ZNF329 | 0 | 1 | 0 | 0 | 0 | 0 | 0 |
| ZNF333 | 0 | 1 | 0 | 0 | 0 | 0 | 0 |
| ZNF335 | 0 | 1 | 0 | 0 | 1 | 0 | 0 |
| ZNF337 | 0 | 1 | 0 | 0 | 0 | 0 | 0 |
| ZNF33A | 0 | 1 | 0 | 0 | 0 | 0 | 0 |
| ZNF341 | 0 | 1 | 0 | 0 | 1 | 0 | 0 |
| ZNF343 | 0 | 1 | 0 | 0 | 0 | 0 | 0 |
| ZNF35 | 0 | 0 | 0 | 1 | 1 | 0 | 0 |
| ZNF350 | 0 | 1 | 0 | 0 | 0 | 0 | 0 |
| ZNF354A | 0 | 0 | 0 | 1 | 0 | 0 | 0 |
| ZNF366 | 0 | 1 | 0 | 0 | 1 | 0 | 0 |
| ZNF382 | 0 | 1 | 0 | 0 | 0 | 0 | 0 |
| ZNF384 | 0 | 1 | 1 | 0 | 1 | 1 | 0 |
| ZNF391 | 0 | 1 | 0 | 0 | 1 | 0 | 0 |
| ZNF394 | 0 | 1 | 0 | 0 | 0 | 0 | 0 |
| ZNF395 | 0 | 1 | 0 | 0 | 1 | 0 | 0 |
| ZNF398 | 0 | 0 | 0 | 0 | 1 | 0 | 0 |
| ZNF414 | 0 | 1 | 0 | 0 | 0 | 0 | 0 |
| ZNF417 | 0 | 0 | 0 | 1 | 0 | 0 | 0 |
| ZNF423 | 0 | 1 | 0 | 0 | 1 | 0 | 0 |
| ZNF430 | 0 | 1 | 0 | 0 | 0 | 0 | 0 |
| ZNF431 | 0 | 1 | 0 | 0 | 0 | 0 | 0 |
| ZNF432 | 0 | 1 | 0 | 0 | 0 | 0 | 0 |
| ZNF44 | 0 | 1 | 0 | 0 | 0 | 0 | 0 |
| ZNF441 | 0 | 1 | 0 | 0 | 0 | 0 | 0 |
| ZNF444 | 0 | 1 | 0 | 0 | 1 | 0 | 0 |
| ZNF446 | 0 | 1 | 0 | 0 | 0 | 0 | 0 |
| ZNF449 | 0 | 1 | 0 | 0 | 1 | 0 | 0 |
| ZNF451 | 0 | 1 | 0 | 0 | 0 | 0 | 0 |
| ZNF454 | 0 | 0 | 0 | 1 | 0 | 0 | 0 |
| ZNF460 | 0 | 1 | 0 | 1 | 0 | 0 | 0 |
| ZNF467 | 0 | 0 | 0 | 0 | 1 | 0 | 0 |
| ZNF48 | 0 | 1 | 0 | 0 | 1 | 0 | 0 |
| ZNF483 | 0 | 1 | 0 | 0 | 0 | 0 | 0 |
| ZNF485 | 0 | 1 | 0 | 0 | 0 | 0 | 0 |
| ZNF501 | 0 | 1 | 0 | 0 | 0 | 0 | 0 |
| ZNF503 | 0 | 1 | 0 | 0 | 0 | 0 | 0 |
| ZNF511 | 0 | 1 | 0 | 0 | 1 | 0 | 0 |
| ZNF524 | 0 | 0 | 0 | 1 | 0 | 0 | 0 |
| ZNF526 | 0 | 1 | 0 | 0 | 0 | 0 | 0 |
| ZNF530 | 0 | 0 | 0 | 1 | 0 | 0 | 0 |
| ZNF543 | 0 | 1 | 0 | 0 | 0 | 0 | 0 |
| ZNF547 | 0 | 1 | 0 | 0 | 1 | 0 | 0 |
| ZNF549 | 0 | 0 | 0 | 1 | 0 | 0 | 0 |
| ZNF550 | 0 | 1 | 0 | 0 | 0 | 0 | 0 |
| ZNF552 | 0 | 1 | 0 | 0 | 0 | 0 | 0 |
| ZNF554 | 0 | 0 | 0 | 0 | 1 | 0 | 0 |
| ZNF556 | 0 | 1 | 0 | 0 | 0 | 0 | 0 |
| ZNF572 | 0 | 1 | 0 | 0 | 0 | 0 | 0 |
| ZNF574 | 0 | 1 | 0 | 1 | 1 | 0 | 0 |
| ZNF579 | 0 | 1 | 0 | 0 | 0 | 0 | 0 |
| ZNF580 | 0 | 1 | 0 | 0 | 1 | 0 | 0 |
| ZNF589 | 0 | 1 | 0 | 0 | 0 | 0 | 0 |
| ZNF596 | 0 | 1 | 0 | 0 | 0 | 0 | 0 |
| ZNF597 | 0 | 0 | 0 | 0 | 1 | 0 | 0 |
| ZNF598 | 0 | 1 | 0 | 0 | 0 | 0 | 0 |
| ZNF600 | 0 | 1 | 0 | 0 | 1 | 0 | 0 |
| ZNF605 | 0 | 1 | 0 | 0 | 0 | 0 | 0 |
| ZNF607 | 0 | 1 | 0 | 0 | 0 | 0 | 0 |
| ZNF608 | 0 | 1 | 0 | 0 | 0 | 0 | 0 |
| ZNF609 | 0 | 1 | 0 | 0 | 0 | 0 | 0 |
| ZNF610 | 0 | 0 | 0 | 1 | 0 | 0 | 0 |
| ZNF614 | 0 | 1 | 0 | 0 | 1 | 0 | 0 |
| ZNF615 | 0 | 1 | 0 | 0 | 0 | 0 | 0 |
| ZNF616 | 0 | 1 | 0 | 0 | 0 | 0 | 0 |
| ZNF618 | 0 | 0 | 0 | 0 | 1 | 0 | 0 |
| ZNF619 | 0 | 1 | 0 | 0 | 0 | 0 | 0 |
| ZNF629 | 0 | 1 | 0 | 0 | 0 | 0 | 0 |
| ZNF639 | 0 | 1 | 0 | 0 | 1 | 0 | 0 |
| ZNF644 | 0 | 1 | 0 | 0 | 1 | 0 | 0 |
| ZNF646 | 0 | 1 | 0 | 0 | 0 | 0 | 0 |
| ZNF652 | 0 | 0 | 0 | 0 | 1 | 0 | 0 |
| ZNF654 | 0 | 1 | 0 | 0 | 1 | 0 | 0 |
| ZNF660 | 0 | 1 | 0 | 0 | 1 | 0 | 0 |
| ZNF667 | 0 | 0 | 0 | 1 | 0 | 0 | 1 |
| ZNF674 | 0 | 1 | 0 | 0 | 0 | 0 | 0 |
| ZNF678 | 0 | 1 | 0 | 0 | 0 | 0 | 0 |
| ZNF680 | 0 | 0 | 0 | 1 | 0 | 0 | 0 |
| ZNF687 | 0 | 1 | 0 | 0 | 1 | 0 | 0 |
| ZNF691 | 0 | 1 | 0 | 0 | 0 | 0 | 0 |
| ZNF692 | 0 | 1 | 0 | 1 | 1 | 0 | 0 |
| ZNF697 | 0 | 1 | 0 | 0 | 0 | 0 | 0 |
| ZNF7 | 0 | 1 | 0 | 0 | 0 | 0 | 0 |
| ZNF701 | 0 | 0 | 0 | 1 | 1 | 0 | 0 |
| ZNF703 | 0 | 1 | 0 | 0 | 0 | 0 | 0 |
| ZNF709 | 0 | 1 | 0 | 0 | 0 | 0 | 0 |
| ZNF71 | 0 | 1 | 0 | 0 | 0 | 0 | 0 |
| ZNF710 | 0 | 1 | 0 | 0 | 0 | 0 | 0 |
| ZNF711 | 0 | 1 | 0 | 0 | 0 | 0 | 0 |
| ZNF737 | 0 | 1 | 0 | 0 | 0 | 0 | 0 |
| ZNF740 | 0 | 1 | 0 | 0 | 1 | 0 | 0 |
| ZNF750 | 0 | 1 | 0 | 0 | 0 | 0 | 0 |
| ZNF76 | 0 | 0 | 0 | 1 | 1 | 1 | 0 |
| ZNF761 | 0 | 1 | 0 | 0 | 0 | 0 | 0 |
| ZNF766 | 0 | 1 | 0 | 0 | 0 | 0 | 0 |
| ZNF770 | 0 | 1 | 0 | 1 | 0 | 0 | 0 |
| ZNF772 | 0 | 1 | 0 | 0 | 0 | 0 | 0 |
| ZNF773 | 0 | 1 | 0 | 0 | 0 | 0 | 0 |
| ZNF776 | 0 | 1 | 0 | 0 | 0 | 0 | 0 |
| ZNF777 | 0 | 1 | 0 | 0 | 0 | 0 | 0 |
| ZNF778 | 0 | 0 | 0 | 0 | 1 | 0 | 0 |
| ZNF782 | 0 | 1 | 0 | 0 | 0 | 0 | 0 |
| ZNF784 | 0 | 1 | 0 | 0 | 0 | 0 | 0 |
| ZNF786 | 0 | 1 | 0 | 0 | 0 | 0 | 0 |
| ZNF792 | 0 | 1 | 0 | 0 | 1 | 0 | 0 |
| ZNF800 | 0 | 1 | 0 | 0 | 0 | 0 | 0 |
| ZNF816 | 0 | 1 | 0 | 1 | 0 | 0 | 0 |
| ZNF83 | 0 | 0 | 0 | 0 | 1 | 0 | 0 |
| ZNF839 | 0 | 1 | 0 | 0 | 0 | 0 | 0 |
| ZNF843 | 0 | 1 | 0 | 0 | 1 | 0 | 0 |
| ZNF850 | 0 | 1 | 0 | 0 | 0 | 0 | 0 |
| ZNF865 | 0 | 1 | 0 | 0 | 0 | 0 | 0 |
| ZNF883 | 0 | 1 | 0 | 0 | 0 | 0 | 0 |
| ZNF891 | 0 | 1 | 0 | 0 | 0 | 0 | 0 |
| ZNF93 | 0 | 0 | 0 | 1 | 0 | 0 | 1 |
| ZSCAN12 | 0 | 1 | 0 | 0 | 0 | 0 | 0 |
| ZSCAN16 | 0 | 0 | 0 | 0 | 1 | 0 | 0 |
| ZSCAN2 | 0 | 0 | 0 | 0 | 1 | 0 | 0 |
| ZSCAN21 | 0 | 1 | 0 | 0 | 0 | 0 | 0 |
| ZSCAN22 | 0 | 0 | 0 | 0 | 1 | 0 | 0 |
| ZSCAN29 | 0 | 0 | 0 | 0 | 1 | 0 | 0 |
| ZSCAN30 | 0 | 1 | 0 | 0 | 1 | 0 | 0 |
| ZSCAN31 | 0 | 1 | 0 | 0 | 0 | 0 | 0 |
| ZSCAN32 | 0 | 1 | 0 | 0 | 0 | 0 | 0 |
| ZSCAN5A | 0 | 1 | 0 | 0 | 1 | 0 | 0 |
| ZSCAN5C | 0 | 1 | 0 | 0 | 0 | 0 | 0 |
| ZSCAN9 | 0 | 1 | 0 | 0 | 1 | 0 | 0 |
| ZXDB | 0 | 1 | 0 | 0 | 1 | 0 | 0 |
| ZXDC | 0 | 1 | 0 | 0 | 1 | 0 | 0 |
