# Supplementary material for: Inhibition of Iron Death by Lycium barbarum Polysaccharides Ameliorates Myocardial Injury in Sepsis: A Pharmacological Mechanism Study Based on the NRF2/HO‐1 Pathway
Source: Food Sci Nutr. 2025 Sep 17;13(9):e70835. doi: 10.1002/fsn3.70835 (PMC12441308; doi:10.1002/fsn3.70835)
Supplement: Supplementary file 3 — Table S3: fsn370835‐sup‐0003‐TableS3.docx. [file FSN3-13-e70835-s004.docx]

| Myocardial Inflammation HE Staining Pathological Section Scoring Form | | |
| --- | --- | --- |
| Level | 0 | No lesion (Not explicitly described, used as baseline control) |
|  | I | Subendocardial focal lesions |
|  | II | Extensive myocardial focal lesions |
|  | III | Widespread confluent myocardial lesions |
|  | IV | Myocardial degeneration and necrosis |

|  | review1 | review2 |
| --- | --- | --- |
| Normal | Based on the examination of the myocardial tissue sections, the overall myocardial tissue appears normal, with no evident morphological alterations in the cardiomyocytes. Therefore, it is graded as Level 0. | Based on the examination of the myocardial tissue sections, the cardiomyocytes exhibit good morphology, presenting the appearance characteristic of normal myocardial cells. Therefore, they are rated as Level 0. |
| LPS | This section shows swelling of muscle fibers, accompanied by multiple hemorrhages and structural damage, therefore it is rated as Level III. | This section exhibits nuclear pyknosis in multiple cardiomyocytes, although the area of necrosis is not extensive. Overall, it should be classified as GradeIV. |
| LPS+LBP | This section shows partial damage to the myocardium, with some cardiomyocytes exhibiting obvious necrosis. However, the overall fibrous morphology of the myocardial tissue remains normal. Therefore, it is rated as Level I. | The overall morphology of this section is good, with some cardiomyocytes showing necrosis and a small amount of myocardial tissue exhibiting significant bleeding. Overall, it is rated as Grade I. |
