# Supplementary material for: Inhibition of Iron Death by Lycium barbarum Polysaccharides Ameliorates Myocardial Injury in Sepsis: A Pharmacological Mechanism Study Based on the NRF2/HO‐1 Pathway
Source: Food Sci Nutr. 2025 Sep 17;13(9):e70835. doi: 10.1002/fsn3.70835 (PMC12441308; doi:10.1002/fsn3.70835)
Supplement: Supplementary file 4 — Table S4: fsn370835‐sup‐0004‐TableS4.docx. [file FSN3-13-e70835-s001.docx]

| b-actin | GPX4 | HO-1 | SLC7A11 | NRF2 | ACSL4 |
| --- | --- | --- | --- | --- | --- |
| 312798 | 540497 | 352611 | 370581 | 360225 | 215014 |
| 281813 | 250014 | 152781 | 313501 | 154565 | 602452 |
| 284182 | 334699 | 358357 | 378381 | 425685 | 375190 |

**Mouse Western Blot (WB) Results Statistical Table**

| b-actin | ACSL4 | HO-1 | SLC7A11 | GOX4 | NRF2 |
| --- | --- | --- | --- | --- | --- |
| 258831 | 0.881911 | 1.440596 | 2.13729 | 1.371003 | 1.476906 |
| 276145 | 1.510326 | 0.527411 | 1.075258 | 0.740281 | 0.586721 |
| 328660 | 0.644024 | 0.93046 | 1.240352 | 1.339637 | 1.385526 |

**H9C2 Western Blot (WB) Results Statistical Table**

| Mouse | SLC7A11 | | | NRF2 | | HO-1 | | GPX4 | | ACSL4 | |
| --- | --- | --- | --- | --- | --- | --- | --- | --- | --- | --- | --- |
|  | Sample | Target **Ct** | Ref **Ct** | Target **Ct** | Ref **Ct** | Target **Ct** | Ref **Ct** | Target **Ct** | Ref **Ct** | Target **Ct** | Ref **Ct** |
|  | mock | 19.17 | 6.83 | 18.33 | 7.12 | 19.93 | 6.95 | 18.31 | 7.11 | 17.21 | 7.05 |
|  |  | 19.06 | 6.95 | 18.47 | 7.09 | 19.95 | 7.03 | 18.53 | 7.14 | 17.26 | 7.01 |
|  |  | 18.96 | 6.96 | 18.47 | 7.21 | 18.74 | 7.13 | 18.69 | 6.95 | 17.31 | 7.11 |
|  | LPS | 20.07 | 7.15 | 19.72 | 7.17 | 20.42 | 7.02 | 19.31 | 6.97 | 16.17 | 7.03 |
|  |  | 20.02 | 7.11 | 19.56 | 7.23 | 20.49 | 6.89 | 19.37 | 6.99 | 16.31 | 6.96 |
|  |  | 20.11 | 7.08 | 19.68 | 7.14 | 20.47 | 6.91 | 19.38 | 7.06 | 16.35 | 7.02 |
|  | LPS+LBP | 19.11 | 6.93 | 18.17 | 6.96 | 19.63 | 7.05 | 18.61 | 7.13 | 17.36 | 7.07 |
|  |  | 19.17 | 7.04 | 18.55 | 6.99 | 19.69 | 7.13 | 18.63 | 7.05 | 17.39 | 6.99 |
|  |  | 19.26 | 7.09 | 18.56 | 7.08 | 19.72 | 7.03 | 18.56 | 6.98 | 17.47 | 7.13 |
| H9C2 | mock | 23.56 | 10.11 | 26.63 | 10.32 | 19.4 | 10.3 | 22.11 | 8.8 | 21.36 | 8.96 |
|  |  | 23.47 | 10.07 | 26.95 | 10.24 | 19.59 | 10.22 | 22.06 | 8.98 | 20.97 | 9.15 |
|  |  | 23.65 | 9.95 | 26.87 | 10.19 | 19.43 | 10.35 | 22.04 | 9.03 | 20.93 | 9.23 |
|  | LPS | 24.43 | 10.15 | 27.53 | 10.11 | 20.82 | 10.06 | 23.06 | 9.15 | 19.65 | 8.89 |
|  |  | 24.74 | 10.26 | 27.61 | 9.97 | 20.47 | 10.11 | 22.96 | 9.1 | 19.71 | 9.02 |
|  |  | 24.53 | 10.06 | 27.74 | 10.06 | 20.75 | 10.17 | 23.45 | 9.15 | 19.74 | 9.11 |
|  | LPS+LBP | 23.71 | 10.16 | 26.59 | 9.89 | 19.07 | 9.89 | 22.61 | 9.47 | 21.31 | 9.23 |
|  |  | 23.66 | 9.93 | 26.41 | 10.15 | 19.24 | 10.07 | 22.53 | 9.41 | 21.11 | 9.06 |
|  |  | 23.93 | 10.11 | 26.74 | 10.17 | 19.05 | 9.74 | 22.47 | 9.22 | 21.19 | 8.99 |

**PCR Ct Value Table (Target and Reference Genes)**
